# Physiologically based pharmacokinetic modeling supports investigation of potential drug-drug interactions in the pre- and early post-hematopoietic stem cell transplantation stages

**DOI:** 10.3389/fphar.2025.1578643

**Published:** 2025-05-02

**Authors:** Peile Wang, Jingli Lu, Jing Yang

**Affiliations:** ^1^ Department of Pharmacy, The First Affiliated Hospital of Zhengzhou University, Zhengzhou, China; ^2^ Henan Key Laboratory of Precision Clinical Pharmacy, Zhengzhou University, Zhengzhou, China; ^3^ Henan Engineering Research Center for Application and Translation of Precision Clinical Pharmacy, Zhengzhou University, Zhengzhou, China

**Keywords:** HSCT, PBPK modeling, DDIs, pharmacokinetics, personalized dosing

## Abstract

**Introduction:**

Drug-drug interactions (DDIs) are an important issue in medication safety and a potential cause of adverse drug events in the pre- and early post-hematopoietic stem cell transplantation (HSCT). This study introduced a physiologically based pharmacokinetic (PBPK) modeling platform to evaluate complex DDIs in these critical stages and to optimize dosing for personalized treatment.

**Methods:**

PBPK models were developed using a bottom-up with middle-out approach and executed with PK-Sim^®^ software. Model validation required that predicted PK values fall within a twofold range of observed data. Then, the validated model was used to simulate alternative dosing regimens to achieve target therapeutic levels.

**Results:**

PBPK models were developed and evaluated for 13 drugs commonly used in HSCT, including cyclosporine, tacrolimus, sirolimus, busulfan, phenytoin, voriconazole, posaconazole, itraconazole, fluconazole, letermovir, fosaprepitant, aprepitant, and omeprazole. Simulation results indicated marked DDIs in the pre- and early post-HSCT phases, particularly involving cyclosporine and phenytoin. Several drugs notably increased cyclosporine concentrations, while phenytoin substantially reduced the exposure to other medications.

**Conclusion:**

This PBPK modeling platform provides a robust tool for identifying and mitigating DDIs in the pre- and early post-HSCT phases. By enabling the optimization of treatment regimens, this model serves as a valuable tool for improving drug safety and therapeutic outcomes for patients with HSCT.

## 1 Introduction

**FIGURE 1:**
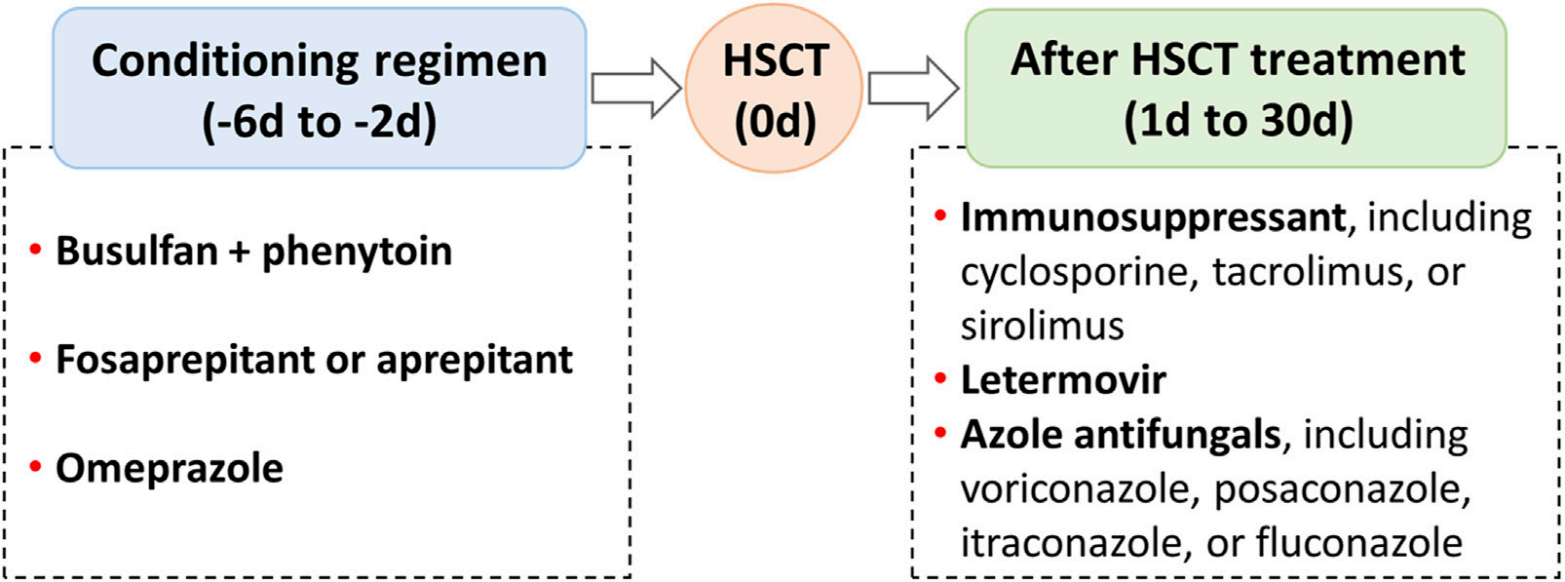
Regimens that may produce drug-drug interactions in the pre- and early post-hematopoietic stem cell transplantation (HSCT).

Hematopoietic stem cell transplantation (HSCT) has become an important treatment option for patients with defined congenital or acquired disorders of the hematopoietic system (Gratwohl et al., 2010). During the pre- (day −6 to day −2) and early post- (day 2 to day 30) HSCT phases, patients often receive complex regimens, including chemotherapeutic, immunosuppressive, and antimicrobial agents (Figure 1; Palmer et al., 2016; Bolaños-Meade et al., 2019; Groll et al., 2021; Ljungman et al., 2025). Certain combinations may produce drug-drug interactions (DDIs), leading to undesirable adverse outcomes and increased drug toxicity (Glotzbecker et al., 2012; Secoli et al., 2015; Pejčić et al., 2019). This concern is particularly relevant for medications with a narrow therapeutic index, such as busulfan, voriconazole, cyclosporine, tacrolimus, and sirolimus (Table 1), which are more prone to unanticipated DDIs when co-administered with enzyme inhibitors or inducers (Palmer et al., 2016; Bolaños-Meade et al., 2019; Groll et al., 2021). In recent years, the clinical use of some new drugs (e.g., fosaprepitant and letermovir) has further increased the complexity of DDIs, so an accurate and reliable model for predicting DDIs is required for dose optimization (Patel et al., 2017; Kim, 2018).

**TABLE 1:** Summary of potential drug-drug-interactions in patients with HSCT and therapeutic target.

Drugs	Major metabolism and transport pathways^a^	Pharmacogenomics (CPIC)^b^	Mechanism^a^	Therapeutic target
Busulfan	GST	—	—	AUC of 900–1,350 μM min
Phenytoin	CYP2C9, CYP2C19	CYP2C9	A strong hepatic drug-metabolizing enzymes inducer	C_min_ of 10–20 μg/mL for epilepticus
Voriconazole	CYP2C19, CYP2C9, CYP3A4	CYP2C19	A strong CYP2C19 and CYP3A4 inhibitor	C_min_ of 1–5 μg/mL
Posaconazole	UGT	—	A strong CYP3A and P-gp inhibitor	C_min_ of ≥0.7 μg/mL
Itraconazole	CYP3A4	—	A strong CYP3A4 inhibitor; a P-gp and BCRP inhibitor	C_min_ of ≥0.5 μg/mL
Fluconazole	Cleared primarily by renal excretion	—	A strong CYP2C19 inhibitor; a moderate CYP2C9 and CYP3A4 inhibitor	—
Letermovir	CYP3A, CYP2D6, P-gp, UGT1A1, UGT1A3, OATP1B1, OATP1B3	—	A CYP3A, CYP2C8, OATP1B1, OATP1B3, P-gp, BCRP, BSEP, and MRP inhibitor; a CYP3A, CYP2C9, and CYP2C19 inducer	—
Fosaprepitant	Converted to aprepitant; CYP3A4, CYP1A2, CYP2C19	—	A weak CYP3A4 inhibitor	—
Aprepitant	CYP3A4, CYP1A2, CYP2C19	—	A weak-to-moderate CYP3A4 inhibitor; a CYP3A4 and CYP2C9 inducer	—
Omeprazole	CYP2C19, CYP3A4	CYP2C19	A moderate CYP3A and CYP2C19 inhibitor	—
Cyclosporine	CYP3A, P-gp	—	A CYP3A4 and multiple drug efflux transporters (e.g., P-gp) inhibitor	C_min_ of 200–400 ng/mL
Tacrolimus	CYP3A	CYP3A5	A CYP3A4 and CYP3A5 inhibitor	C_min_ of 5–15 ng/mL
Sirolimus	CYP3A4, P-gp	—	—	C_min_ of 3–8 ng/mL

^a^ from drugs.com website.

^b^ from Clinical Pharmacogenetics Implementation Consortium (CPIC) guidelines; CYP, cytochrome P450; GST, glutathione S-transferase; UGT, UDP-glucuronosyltransferase; OATP, organic anion transporting polypeptides; P-gp, P-glycoprotein; BCRP, breast cancer resistance protein; BSEP, bile salt export pump; MRP, multidrug resistance-associated protein; AUC, the area under the curve; C_min_, trough concentration.

Currently, clinically relevant DDIs can be queried through online and offline interaction checkers, such as Drugs, Lexi-Interact, and ePocrates, but the available clinical dosage suggestions are limited (Storelli et al., 2018). However, model-informed precise dosing can leverage pharmacokinetic (PK) models to tailor individualized dosing (Darwich et al., 2021). Therefore, an increasing number of studies have applied physiologically based PK (PBPK) models to predict drug PK profiles by integrating drug property parameters with physiological parameters of the organism. Unlike interaction checkers, PBPK models are more prominent in describing time-variable concentrations of drugs and predicting DDI in the early stages of drug development and complex clinical scenarios, such as simultaneous inhibition and induction of enzymes and transporters, complex transporter-enzyme interplay, and genetic variations of cytochrome P450 (CYP) enzymes (Vijaywargi et al., 2023; Foti, 2025). Accordingly, PBPK models have been widely used in drug discovery and development areas, including DDIs, organ injury, pediatrics, drug-gene interactions, disease impact, and food effects (Sun et al., 2024).

To date, several studies have developed PBPK models to predict the PK profiles of busulfan, phenytoin, voriconazole, posaconazole, itraconazole, fluconazole, letermovir, omeprazole, cyclosporine, tacrolimus, and sirolimus (Supplementary Table S1). However, due to the different diseases or populations of concern, DDIs caused by common drug combinations in patients with HSCT have rarely been reported, such as busulfan in combination with phenytoin, voriconazole or posaconazole in combination with cyclosporine, etc. Furthermore, no PBPK model for fosaprepitant and aprepitant has been reported. So, there is still a need for further models and more comprehensive DDIs networks.

This study aimed to develop a PBPK modeling platform to investigate the complex DDIs of 13 commonly used drugs in the pre- and early post-HSCT phase, including busulfan, phenytoin, voriconazole, posaconazole, itraconazole, fluconazole, letermovir, omeprazole, fosaprepitant, aprepitant, cyclosporine, tacrolimus, and sirolimus. Furthermore, dose optimization of the above drugs was predicted for personalized treatment.

## 2 Materials and methods

### 2.1 Software

PBPK model development and simulations were performed with PK-Sim^®^ (Version 11.2, Bayer Technology Services, Leverkusen, Germany). Clinical study data from published literature were collected with the semi-automated tool WebPlotDigitizer (Version 4.6, Ankit Rohatgi, Pacifica, CA, United States). For plot generation, GraphPad Prism 8.0.1 (GraphPad Software Inc., San Diego, CA, United States) was used.

### 2.2 PBPK models development and verification

**FIGURE 2:**
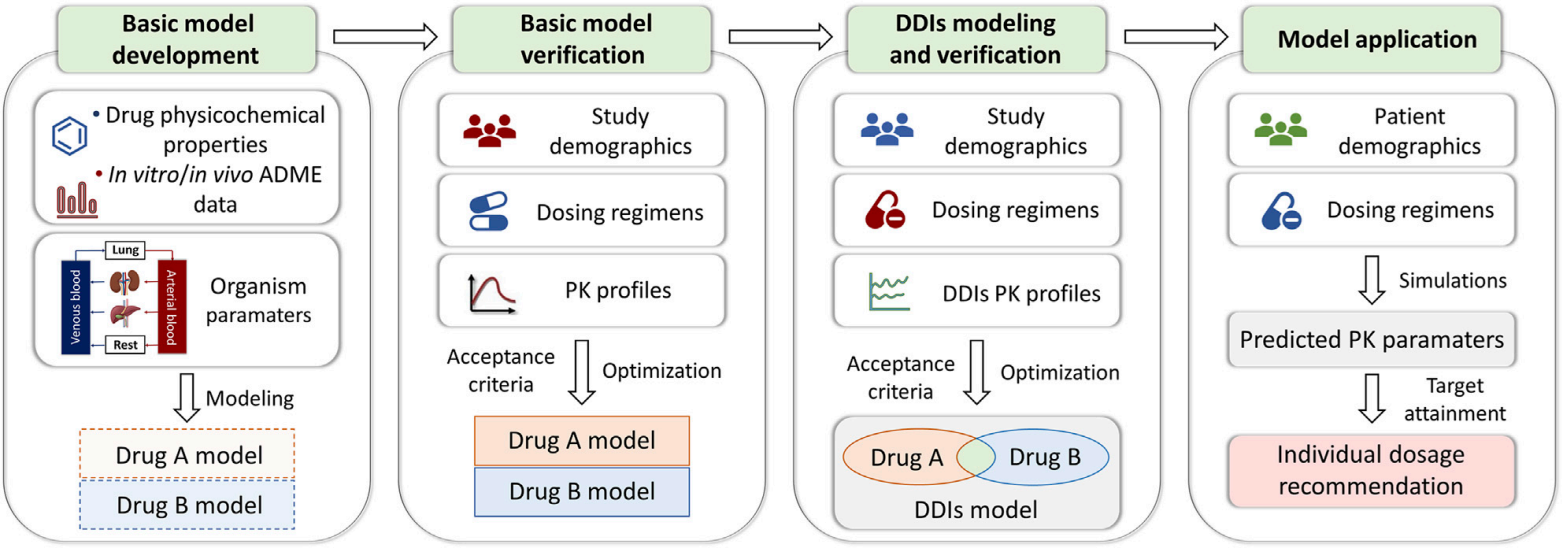
PBPK modeling framework detailing the processes of model development and verification. ADME, absorption, distribution, metabolism, and elimination; PK, pharmacokinetic; DDIs, drug-drug interactions.

PBPK models for 13 drugs were developed based on the bottom-up with middle-out techniques (Sun et al., 2024), and the workflow adopted for model development, verification, and application was illustrated in Figure 2. Briefly, a PBPK modeling of a drug mainly consists of expression profiles, individuals, populations, compounds, formulations, administration protocols, and observed data. Protein expression of enzymes and transporters was implemented using the PK-Sim^®^ database. Individuals or populations used to simulate the different studies were modeled according to the corresponding study reports, with age, weight, height, gender, and ethnicity (Supplementary Material). If the demographic information was not found, a 30-year-old male European was assumed, with the mean weight and height characteristics in the software database. Other building blocks required an extensive literature search to gain information about the physicochemical properties and absorption, distribution, metabolism, and excretion processes of the drug. Meanwhile, plasma concentration-time profiles of intravenous and oral administration in single or multiple doses from healthy individuals or clinical studies were digitized and used for model development and evaluation. Parameters that were not informed from the literature were optimized, fitting the model to the observed PK data in the literature.

The predictive performance of PBPK models was initially evaluated by comparing predicted plasma concentration-time profiles to observed data. The PK parameters assessed were the area under the curve (AUC) and peak plasma concentration (C_max_), which were quantitatively assessed based on a predefined criterion of two-fold range (0.5–2.0) of the predicted/observed ratio.

### 2.3 DDIs network modeling and verification

In the DDIs model, there are five specified types of inhibition, including competitive, uncompetitive, non-competitive, mixed, and irreversible inhibition. Inhibition types for different drugs are based on literature reports, software databases, and optimization. Besides, drug-mediated induction of enzymes and transporters was expressed in terms of the maximum induction effect (Emax) and the concentration supporting half of Emax (EC50).

The performance of the DDI model was evaluated by comparing the predicted to observed victim drug plasma concentration-time profiles when administered alone and during coadministration. The metric to assess the DDI modeling performance was the predicted DDI ratios of AUC and C_max_ (AUC or C_max_ of victim drug during coadministration/AUC or C_max_ of victim drug alone) to the observed DDI ratios of AUC and C_max_. A two-fold error margin (0.5–2.0) was set as the acceptance criterion.

### 2.4 Prospective PBPK DDI scenario for dose optimization

To mimic the clinical setting, the verified model was used to simulate the exposure of drugs that need therapeutic drug monitoring (TDM), such as voriconazole, posaconazole, busulfan, cyclosporine, tacrolimus, and sirolimus. A virtual population of 100 Japanese individuals with the mean weight and height characteristics given in the software database was generated. Subsequently, the fold change in AUC or trough concentration (C_min_) at steady-state was calculated during coadministration to when administered alone. Voriconazole C_min_ of 1–5 μg/mL, posaconazole C_min_ of ≥0.7 μg/mL, itraconazole C_min_ of ≥0.5 μg/mL, busulfan AUC of 900–1,500 μM min, cyclosporine C_min_ of 200–400 ng/mL, tacrolimus C_min_ of 5–15 ng/mL, and sirolimus C_min_ of 3–8 ng/mL are used as the therapeutic index for individualized treatment (Palmer et al., 2016; Bolaños-Meade et al., 2019; Groll et al., 2021).

## 3 Results

### 3.1 PBPK model building and evaluation

The PBPK models of three immunosuppressants (cyclosporine, tacrolimus, and sirolimus), one chemotherapeutic agent and its anticonvulsant prophylactic agent (busulfan and phenytoin), four antifungal agents (voriconazole, posaconazole, itraconazole, and fluconazole), one antiviral agent (letermovir), two antiemetic agents (fosaprepitant and aprepitant), and omeprazole were developed and comprehensively evaluated using the blood concentration-time profiles. Among them, parameters for itraconazole, fluconazole, and omeprazole were taken directly from the PK-Sim^®^ database, and those for voriconazole and tacrolimus were taken directly from the previously established models (Gong et al., 2023; Wang et al., 2024). The rest of drug parameters were gathered from the literature and optimized based on blood concentration-time profiles from different literature, as summarized in Supplementary Table S2.

Model performance was demonstrated by comparison of predicted to observed plasma concentration-time profiles in Supplementary Figures S1–S7, and predicted to observed AUC and C_max_ values was presented in Supplementary Table S3. As a result, the predicted to observed AUC and C_max_ ratios were within 0.53–1.71-fold error.

### 3.2 DDI network modeling

In this study, irreversible inhibition processes of CYP3A4 for voriconazole and tacrolimus and CYP2C19 for omeprazole were modeled using *Ki* (inhibition constant) and *k*_*inact*_ (maximum inactivation rate constant) values (Li et al., 2020; Loer et al., 2023); the competitive inhibition of CYP450, organic anion-transporting peptide (OATP) 1B1/3 and P-glycoprotein (P-gp) for posaconazole, itraconazole, fluconazole, aprepitant, cyclosporine, tacrolimus, and letermovir was modeled using *Ki* values (Gertz et al., 2013; Gerner et al., 2022; Loer et al., 2023); the induction of CYP2C9, CYP2C19, CYP3A4, UDP glucuronosyltransfer (UGT) 1A4 and glutathione S-transferase (GST) A1 for phenytoin was implemented using EC50 and Emax values (Rodriguez-Vera et al., 2023).

The performance of DDI models was evaluated by comparing predicted to observed PK profiles of victim drugs with/without an enzyme or transporter perpetrator coadministration (Supplementary Figures S8–S14). In addition, the predicted to observed DDI AUC ratios and C_max_ ratios of all DDI studies were shown in Supplementary Table S4, with a range of 0.54–1.86 for predicted to observed parameters.

### 3.3 Prospective PBPK DDI scenario for dose optimization

**FIGURE 3:**
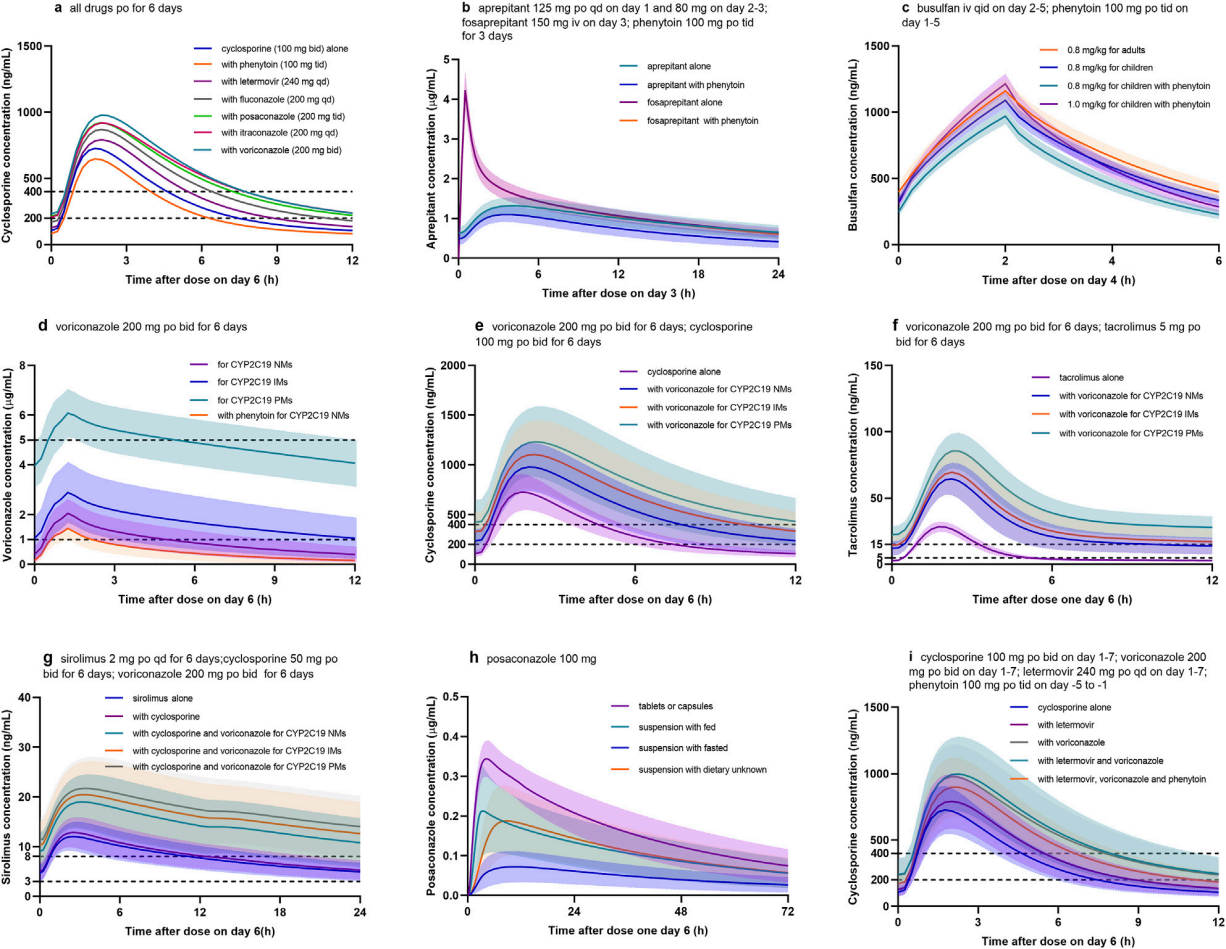
Simulated concentration profiles of drugs used in the pre- and early post HSCT. **(a)** Cyclosporine with different CYP450 inhibitors or inducers; **(b)** aprepitant or fosaprepitant with phenytoin; **(c)** busulfan with phenytoin; **(d)** voriconazole with phenytoin for CYP2C19 normal metabolizers (NM), intermediate metabolizers (IM), and poor metabolizers (PM); **(e)** cyclosporine with voriconazole for different CYP2C19 phenotypes; **(f)** tacrolimus with voriconazole for different CYP2C19 phenotypes; **(g)** sirolimus with voriconazole for different CYP2C19 phenotypes; **(h)** posaconazole for different formulations and dietary; **(i)** cyclosporine with voriconazole, letermovir, and phenytoin.

To show the utility of the model in personalized therapy, dose adaptations for different DDI scenarios were simulated. Plasma concentration-time profiles of victim drug during co-administration of perpetrator drug were simulated and compared to those without perpetrator drug (Figure 3). Notably, the coadministration of voriconazole, itraconazole, posaconazole, fluconazole, or letermovir increased cyclosporine C_min_ of approximately 2.2-, 2.1-, 2.0-, 1.6-, and 1.2-fold, respectively (Table 2). Conversely, coadministration of phenytoin decreased cyclosporine C_min_, aprepitant AUC, fosaprepitant AUC, busulfan AUC, and voriconazole C_min_ by approximately 21.1%, 36.9%, 8.5%, 13.4%, and 51.0%, respectively (Figures 3a–d).

**TABLE 2:** Simulated pharmacokinetic parameters of cyclosporine with different drugs.

Victim drug (cyclosporine)	Perpetrator drug	Predicted C_min_ (ng/mL; mean ± SD)	C_min_ ratio
100 mg po bid	—	104.9 ± 32.2	—
100 mg po bid	phenytoin 100 mg po tid	82.7 ± 26.1	0.8
100 mg po bid	letermovir 240 mg po qd	126.3 ± 49.7	1.2
100 mg po bid	fluconazole 200 mg po qd	169.2 ± 67.6	1.6
100 mg po bid	posaconazole 200 mg po tid	208.6 ± 113.1	2.0
100 mg po bid	itraconazole 200 mg po qd	215.2 ± 119.1	2.1
100 mg po bid	voriconazole 200 mg po bid for CYP2C19 NMs	233.8 ± 147.7	2.2
100 mg po bid	voriconazole 200 mg po bid for CYP2C19 IMs	327.7 ± 193.3	3.1
100 mg po bid	voriconazole 200 mg po bid for CYP2C19 PMs	422.7 ± 227.2	4.0
100 mg po bid	voriconazole 200 mg po bid for CYP2C19 NMs; letermovir 240 mg po qd	240.6 ± 124.6	2.3
100 mg po bid	voriconazole 200 mg po bid for CYP2C19 NMs; letermovir 240 mg po qd; phenytoin 100 mg po tid on day −5 to −1	173.1 ± 76.3	1.7
150 mg po bid	voriconazole 200 mg po bid for CYP2C19 NMs; letermovir 240 mg po qd; phenytoin 100 mg po tid on day −5 to −1	261.3 ± 109.7	2.5

C_min_, minimum plasma concentration on day 6; SD, standard deviation; NMs, normal metabolizers; IMs, intermediate metabolizers; PMs, poor metabolizers.

## 4 Discussion

Over the past decade, the use of PBPK modeling has been rising, and regulatory agencies have issued DDI guidance highlighting the use of such modeling approaches (Sun et al., 2024; Paul et al., 2025). Commonly used PBPK software packages include SimCYP (Certara), GastroPlus (Simulations Plus), and PK-Sim (Open Systems Pharmacology), among which PK-Sim is the only open-source platform and is used for free. Alternatively, FDA reviewers endorsed the predicted results for all three platforms (Sun et al., 2024). This method using in HSCT has several advantages:

Firstly, it allows for the extrapolation of PK behavior of drugs in healthy volunteers to diseased or special populations (e.g., the elderly, children, pregnant women, and patients with hepatic and renal insufficiency). Busulfan is a widely used chemotherapic agent in pediatrics with HSCT. Given the narrow therapeutic index, it is routinely dosed with TDM (Palmer et al., 2016). In this PBPK model, age-dependent enzyme activity was tailored and evaluated with an external dataset (Supplementary Tables S3, S4). The results indicated that at the same dose (0.8 mg/kg), busulfan exposure in children (979.4 ± 76.2 μM min) was lower than that in adults (1,078.8 ± 103.9 μM min). When co-administered with phenytoin, busulfan exposure in children (847.9 ± 68.4 μM min) was even lower (Figure 3c) and below the therapeutic window (AUC of 900–1,350 μM min; Palmer et al., 2016); while when increasing busulfan dose to 1.0 mg/kg, the exposure (1,060.7 ± 95.5 μM min) was sufficient.

Secondly, the model can assess inter-individual variability in a virtual population by incorporating factors, such as age, gender, ethnicity, and genetic polymorphisms. Voriconazole demonstrates wide interpatient variability in serum concentrations due in part to variant *CYP2C19* alleles (Moriyama et al., 2017). In this PBPK model, different CYP2C19 phenotypes were integrated into the model as normal metabolizer (NM), intermediate metabolizer (IM), and poor metabolizer (PM), with reference values for *CYP2C19* expression of 0.76, 0.40, and 0.01 μmol/L, respectively (Wang et al., 2024). As shown in Figure 3d, AUC for CYP2C19 IMs and PMs were 2.3- to 4.0-fold higher than those for CYP2C19 NMs. The standard oral maintenance dose of voriconazole 200 mg twice daily would be sufficient for CYP2C19 IMs and PMs to reach the tentative therapeutic range of 1.0–5.0 μg/mL for C_min_, while 400 mg twice daily might be more suitable for NMs. Meanwhile, the DDIs between voriconazole and three immunosuppressants were also affected by CYP2C19 genotypes (Figures 3e–g). Similarly, tacrolimus exhibited large inter-and intra-individual PK variability, partly due to genetic variations in *CYP3A5* (Birdwell et al., 2015). Accordingly, different CYP3A5 genotypes were integrated into the model as CYP3A5 expressers (*1/*1 or *1/*3) and non-expressers (*3/*3), with reference values of 0.68 and 0.04 μmol/L for CYP3A5 expression in liver tissues, respectively (Loer et al., 2023).

Thirdly, it enables investigation of the effect of factors, such as formulations, administration routes, and food, on drug PK parameters. In terms of posaconazole, the exposure for oral suspension increased 2.5- to 3.0-fold when it was given with a high-fat meal; whereas, exposures for tablets and capsules were not markedly affected by food and were higher than that for oral suspension (Krishna et al., 2012). During the modeling, intestinal permeability and Weibull parameters were adjusted, and the final model examined four scenarios (tablet or capsules, suspension with dietary unknown, suspension with fasted, and suspension with fed) to predict the influence of food intake and formulation on posaconazole PK profile (Supplementary Table S3; Figure 3h).

Fourthly, the model supports the optimization of rational dosing regimens by integrating therapeutics index or pharmacodynamic indicators. Following HSCT, cyclosporine is commonly administered in combination with voriconazole and letermovir, which are both CYP3A4 inhibitors, and the latter is also an inhibitor of P-gp transporter (Table 1). Thus, when co-administrated with voriconazole and letermovir, simulations showed that the steady-state C_min_ of cyclosporine on day 6 increased 2.3-fold compared with cyclosporine (100 mg) alone (Figure 3i; Table 2). Whereas, phenytoin is commonly used as anticonvulsant prophylaxis for busulfan before HSCT. On discontinuation of phenytoin, CYP450 activity may not be fully restored for 7–10 days (Spriet et al., 2010), which may result in decreased concentrations of cyclosporine, voriconazole, and letermovir. Therefore, when phenytoin was administered 5 days before the three drugs combination, simulation showed that cyclosporine C_min_ (173.2 ± 76.3 ng/mL) on day 6 was still lower than that without phenytoin (240.6 ± 124.6 ng/mL) and also below the therapeutic window (C_min_ of 200–400 ng/mL). When increasing cyclosporine dose to 150 mg, the C_min_ (261.3 ± 109.7 ng/mL) met the requirements.

There are several limitations to the present PBPK model. Firstly, the metabolism and DDIs were based on enzyme kinetic parameters observed from *in vitro* experiments, while *in vitro* data may not accurately describe drug changes *in vivo*. This is a common problem with the PBPK model. To ensure the credibility of the final PBPK model, the source of the model parameters was provided, and the current PBPK models were created and refined by combining the bottom-up and middle-out approaches, that is, modifying the parameters to fit the observed data. Secondly, the population parameters for patients with HSCT are currently lacking or challenging to acquire, so this PBPK model does not take into consideration the physiological differences in patients with HSCT. Thirdly, the WHO model evaluation criteria were used in this study, i.e., the predicted-to-observed ratio within a factor of 2 (WHO, 2010). As stated by Guest et al., this strategy results in a potential bias toward successful prediction at lower interaction levels (Guest et al., 2011). For observed DDI ratios of 1 (no interaction), the 2-fold deviation would allow predicted DDI ratios between 0.5 (induction) and 2 (weak to moderate inhibition), which could overstate the DDI performance for weak interactions. Therefore, the methodology proposed by Guest et al. may be more stringent for DDI prediction assessment, accepting a 20% deviation for observed DDI ratios approaching 1. Lastly, there is a lack of clinical validation for prospective DDI scenarios for dose optimization, which needs further research.

Despite its shortcomings, the most meaningful application of PBPK is to obtain the data through prediction in the absence of clinical data and to guide clinical research. In the future, benefiting from the development of software platforms and a more comprehensive understanding of human physiological changes, especially when there are factors, such as disease, that lead to physiological changes, which means that PBPK models will be more widely applied in novel drug development and dose optimization (Sun et al., 2024).

## 5 Conclusion

In conclusion, this study introduced a PBPK modeling platform to predict potential DDIs and their impact on drug exposure in patients in the pre- and early post-HSCT. By enabling the optimization of treatment regimens, this model may serve as a valuable tool for improving drug safety and therapeutic outcomes.

## Data Availability

The original contributions presented in the study are included in the article/Supplementary Material, further inquiries can be directed to the corresponding author.
