# Supplementary material for: Physiologically based pharmacokinetic modeling supports investigation of potential drug-drug interactions in the pre- and early post-hematopoietic stem cell transplantation stages
Source: Front Pharmacol. 2025 May 2;16:1578643. doi: 10.3389/fphar.2025.1578643 (PMC12081248; doi:10.3389/fphar.2025.1578643)
Supplement: Supplementary file 2 [file Supplementaryfile2.docx]

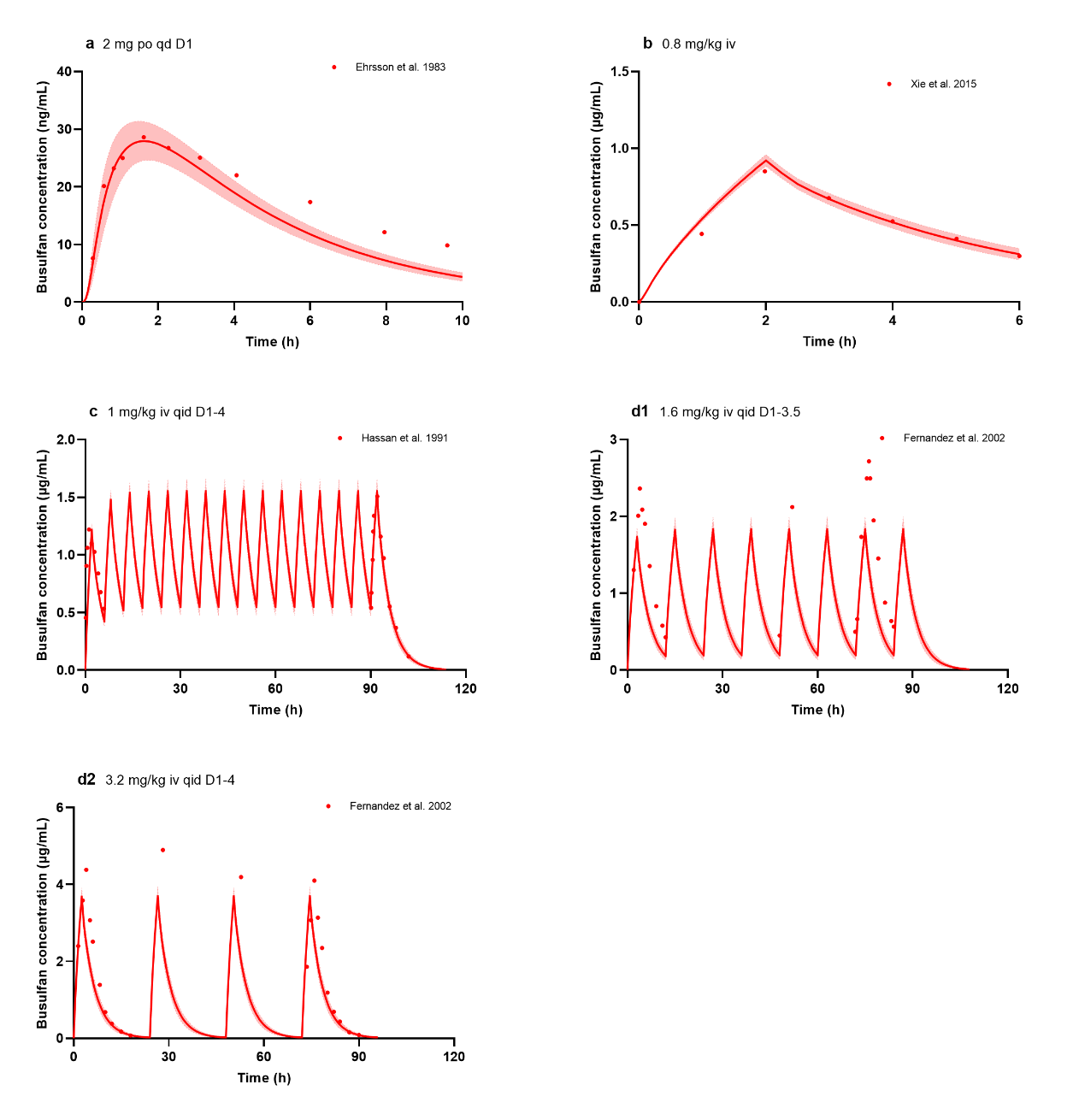


Figure S1. Prediction performance of busulfan pharmacokinetic based on PBPK model.^87,90-92^


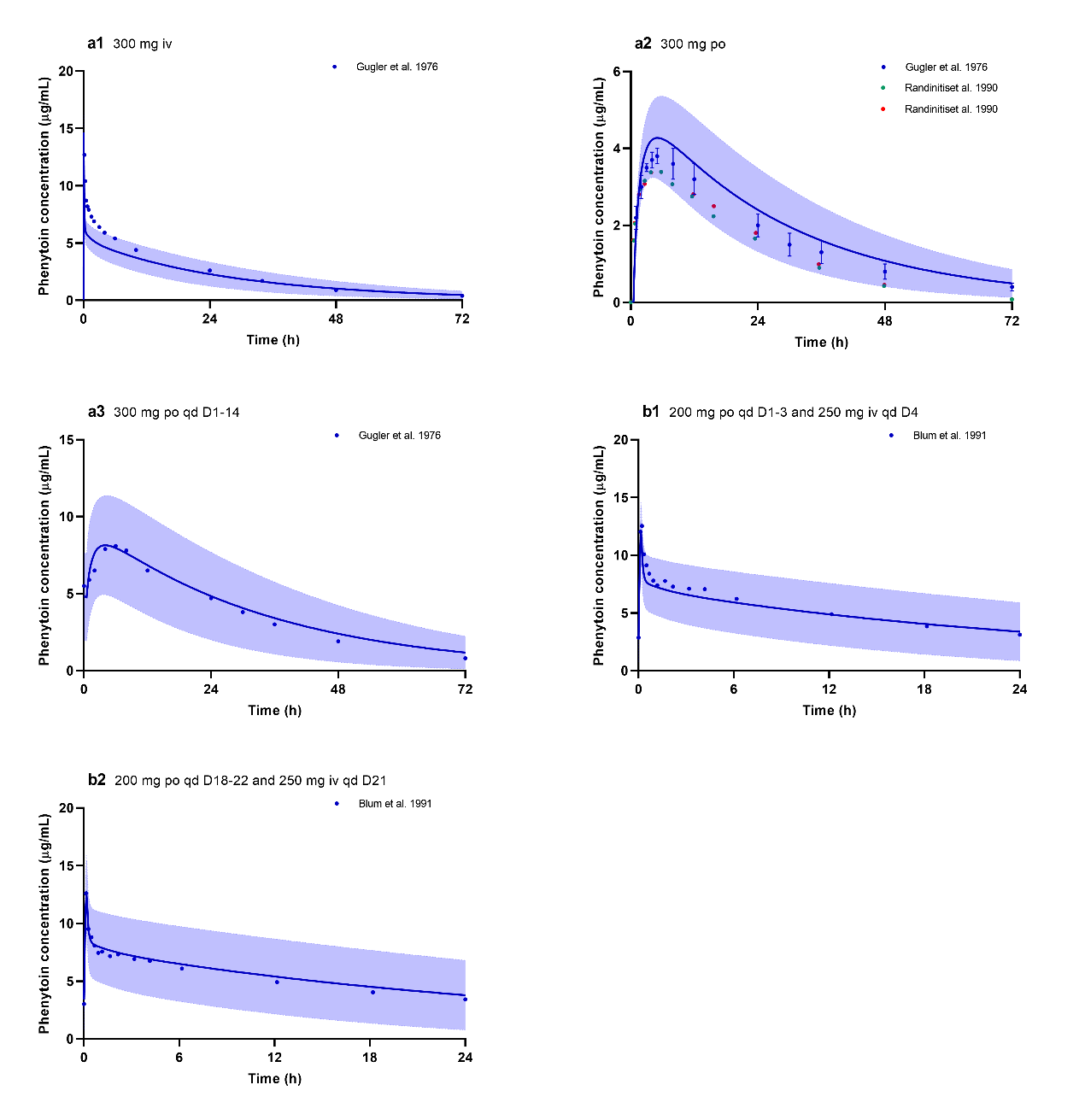


Figure S2. Prediction performance of phenytoin pharmacokinetic based on PBPK model.^94-96^


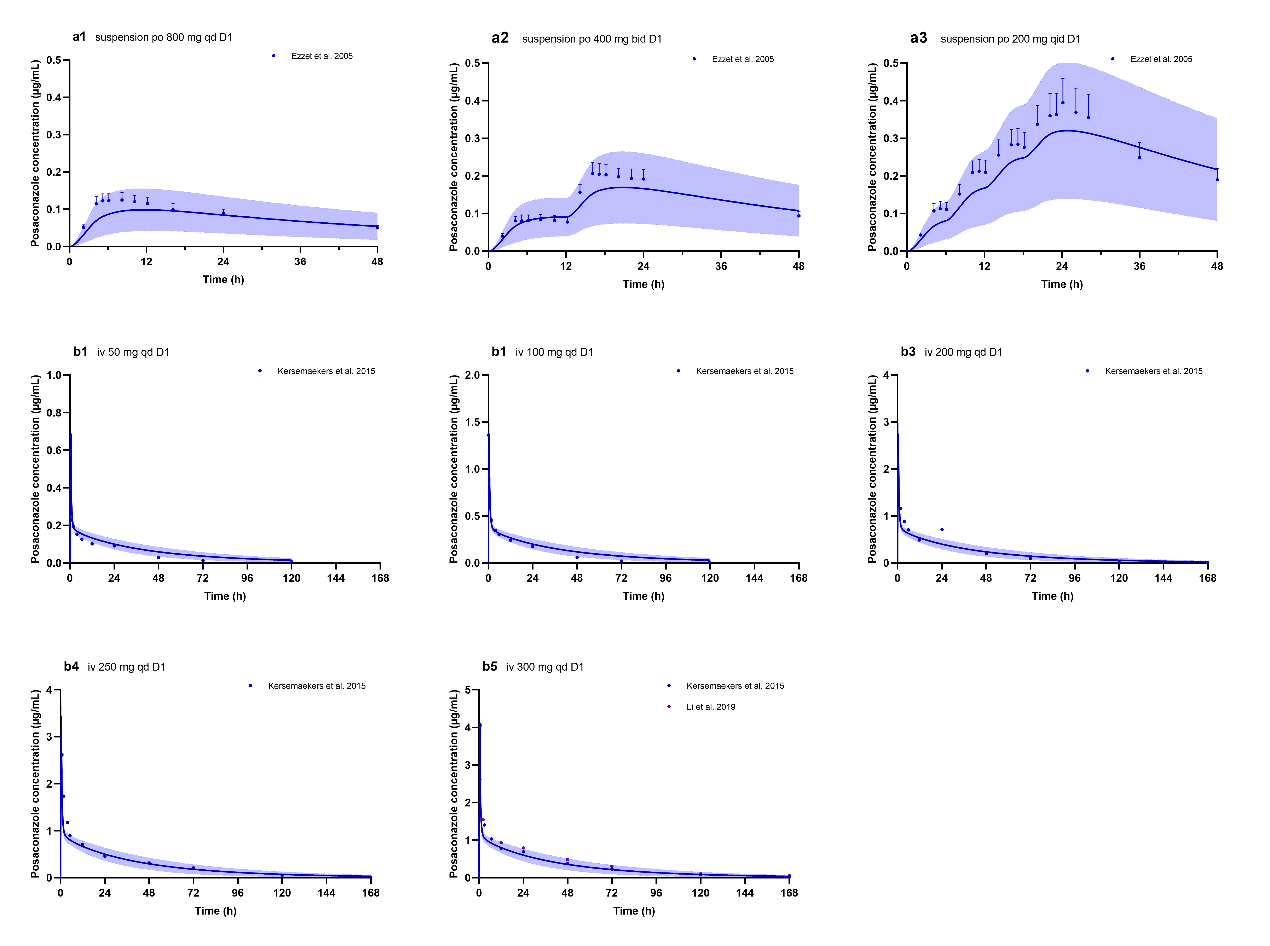


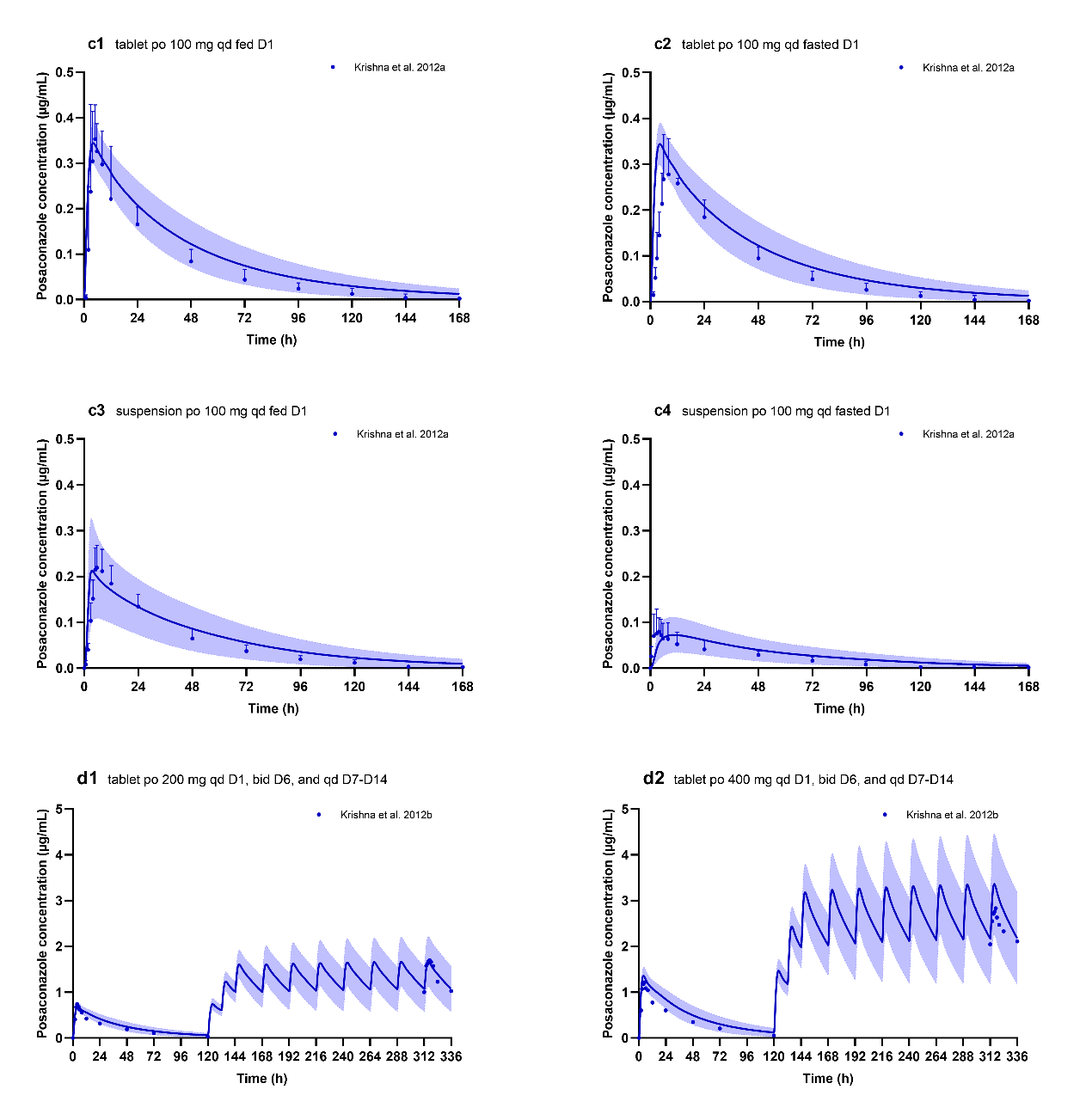


Figure S3. Prediction performance of posaconazole pharmacokinetic based on PBPK model.^101-105^


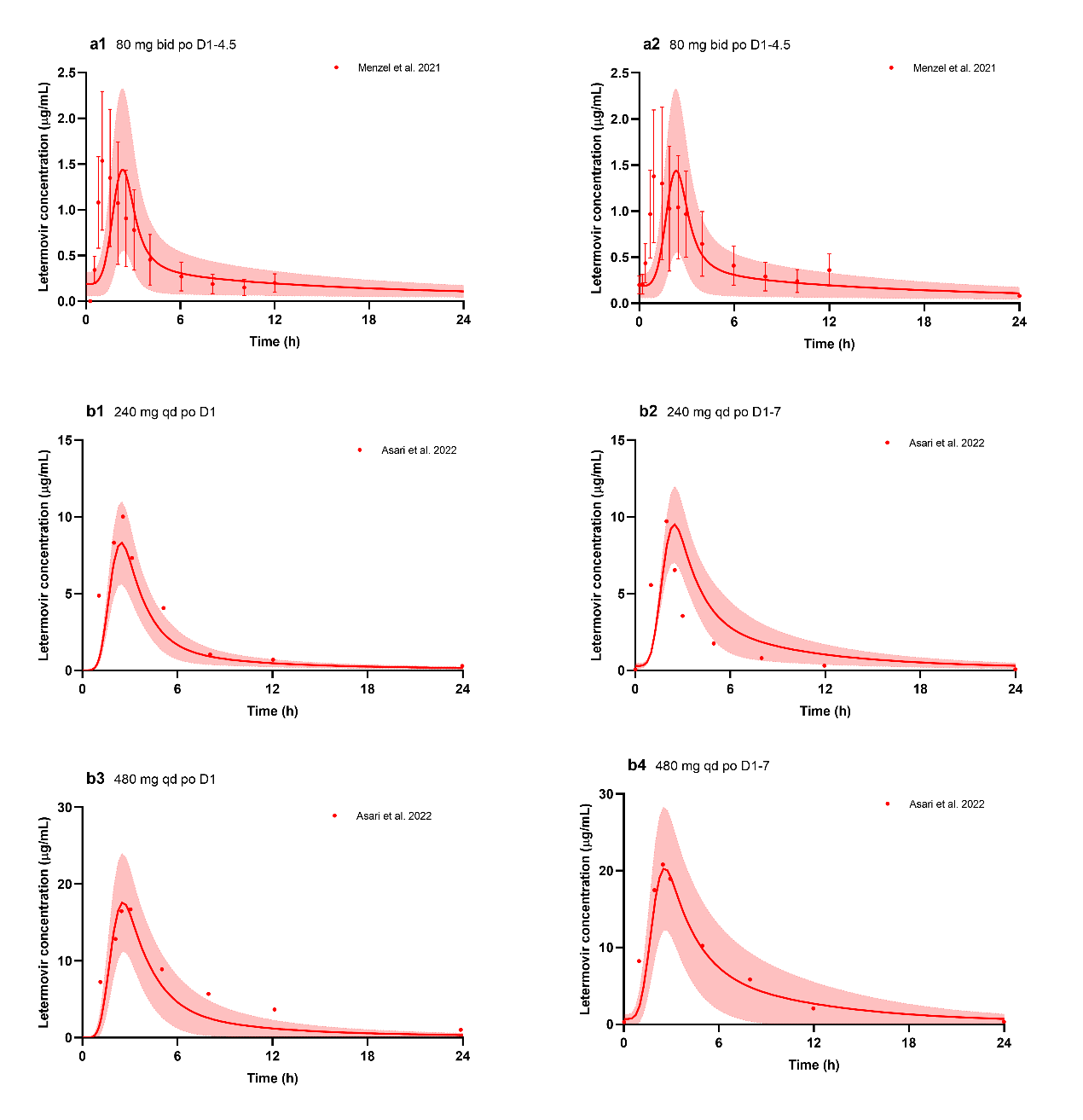


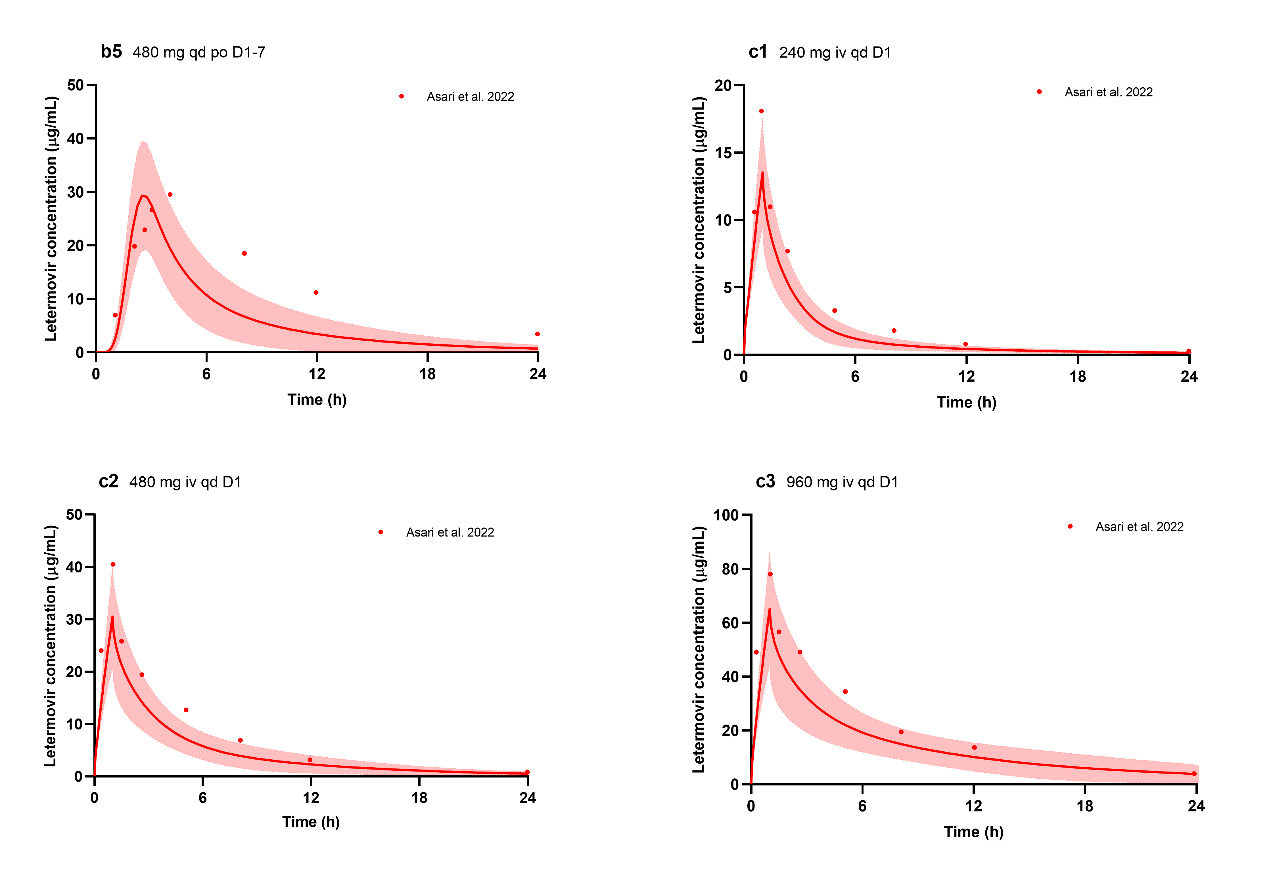


Figure S4. Prediction performance of letermovir pharmacokinetic based on PBPK model.^109,110^


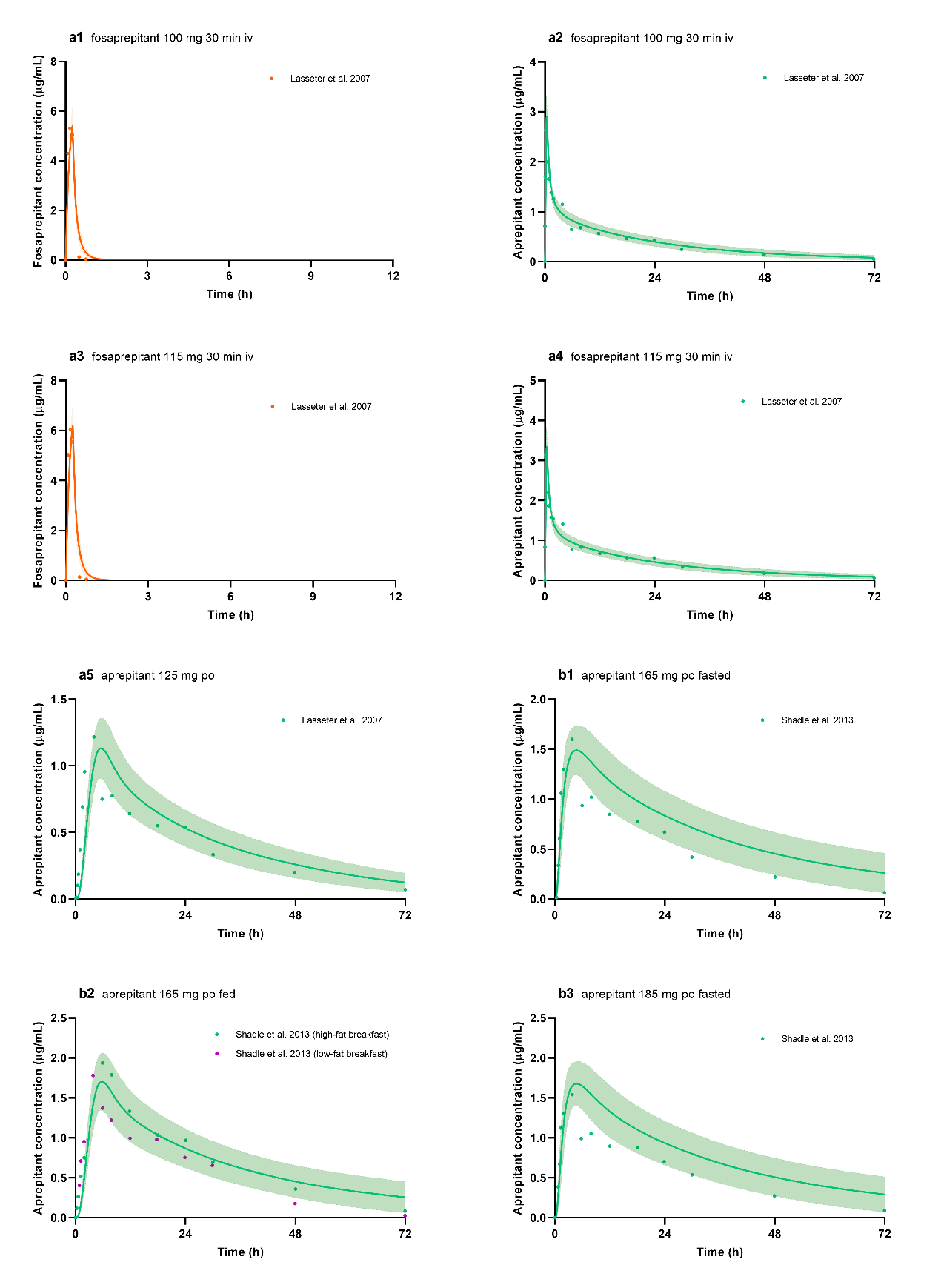

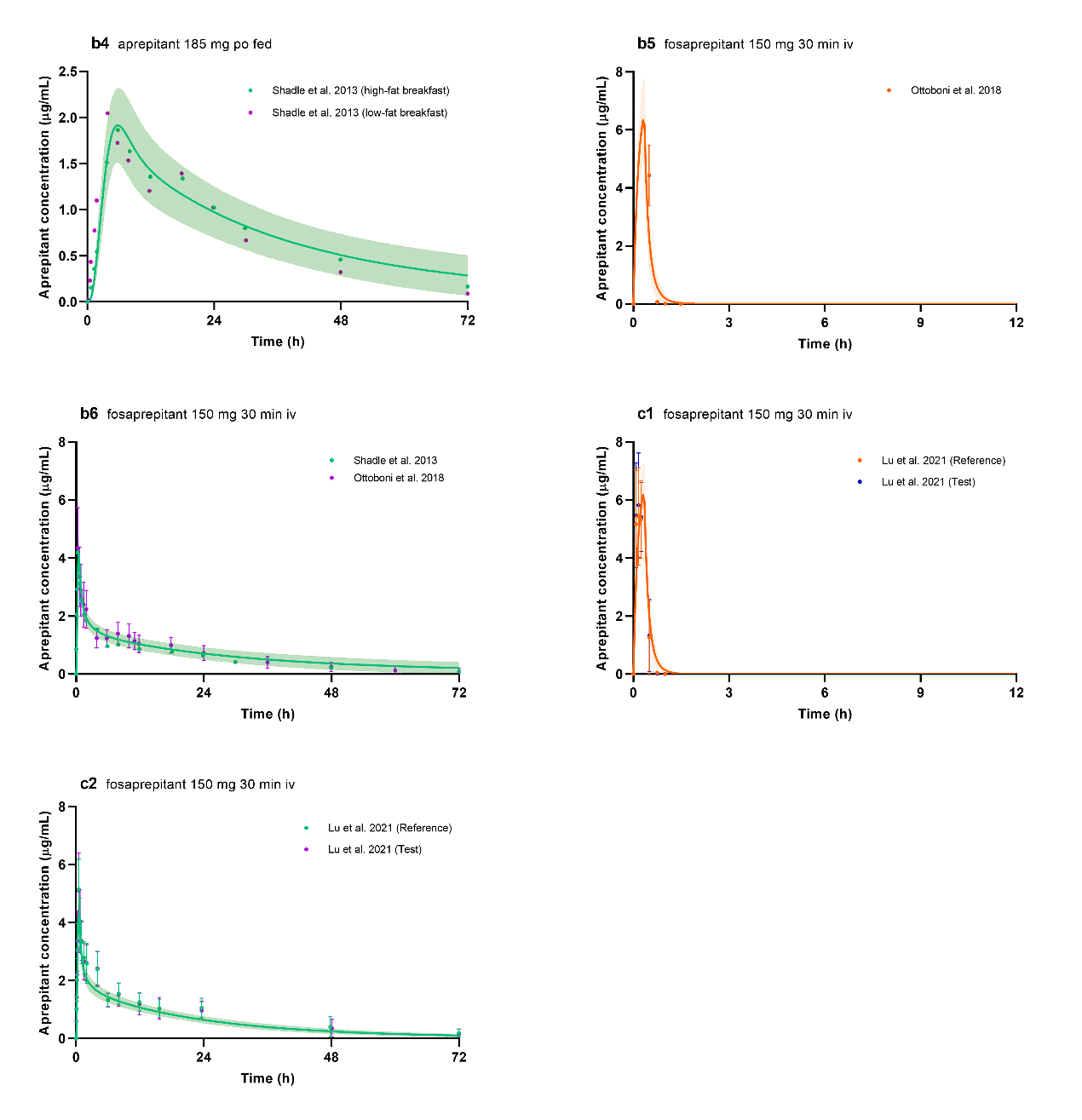


Figure S5. Prediction performance of aprepitant and fosaprepitant pharmacokinetic based on PBPK model.^113-116^


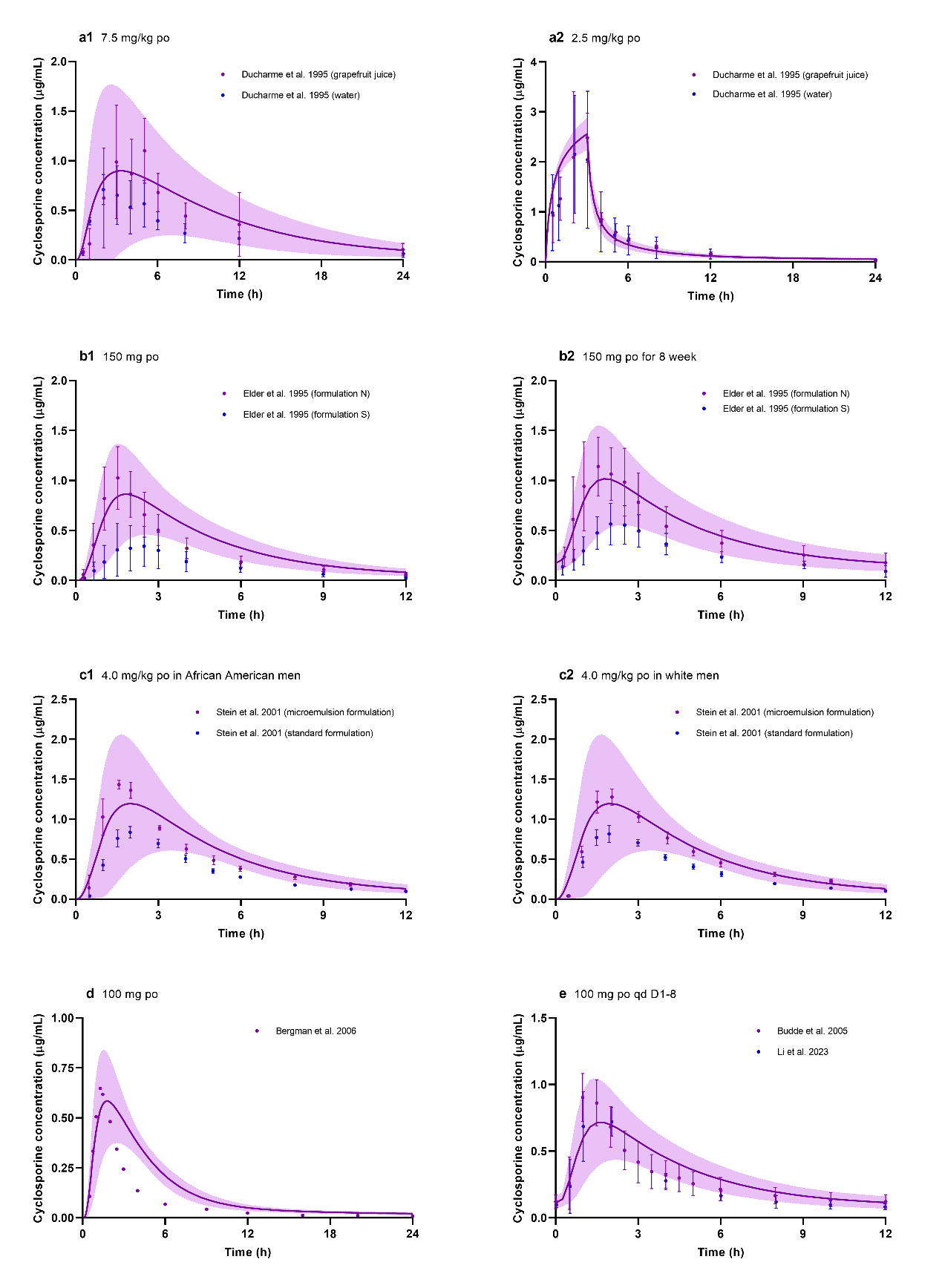
 Figure S6. Prediction performance of cyclosporine pharmacokinetic based on PBPK model.^117-119,121-123^


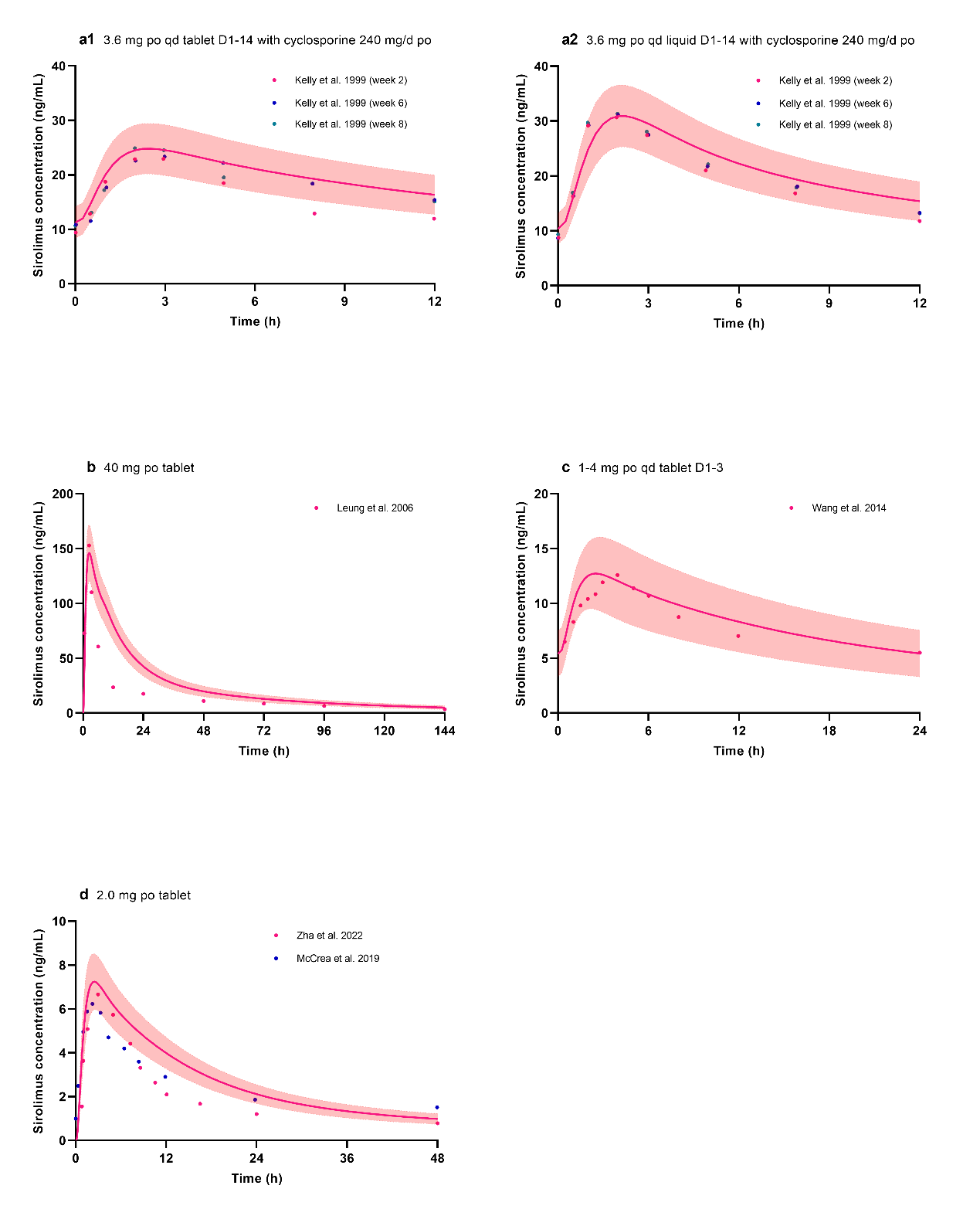


Figure S7. Prediction performance of sirolimus pharmacokinetic based on PBPK model.^107,127-129,131^


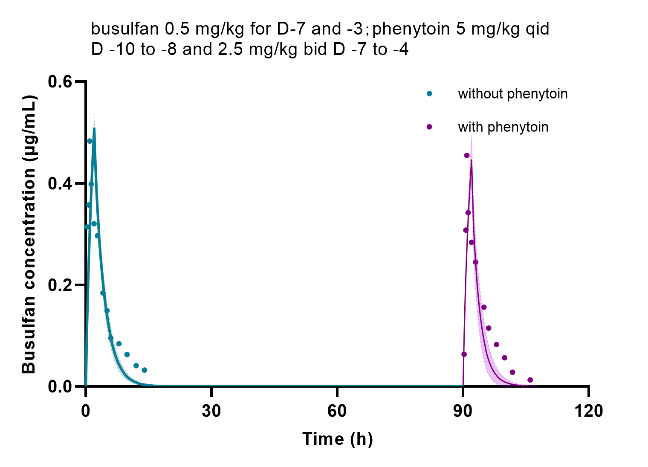


Figure S8. Prediction performance of busulfan DDI based on PBPK model.^132^


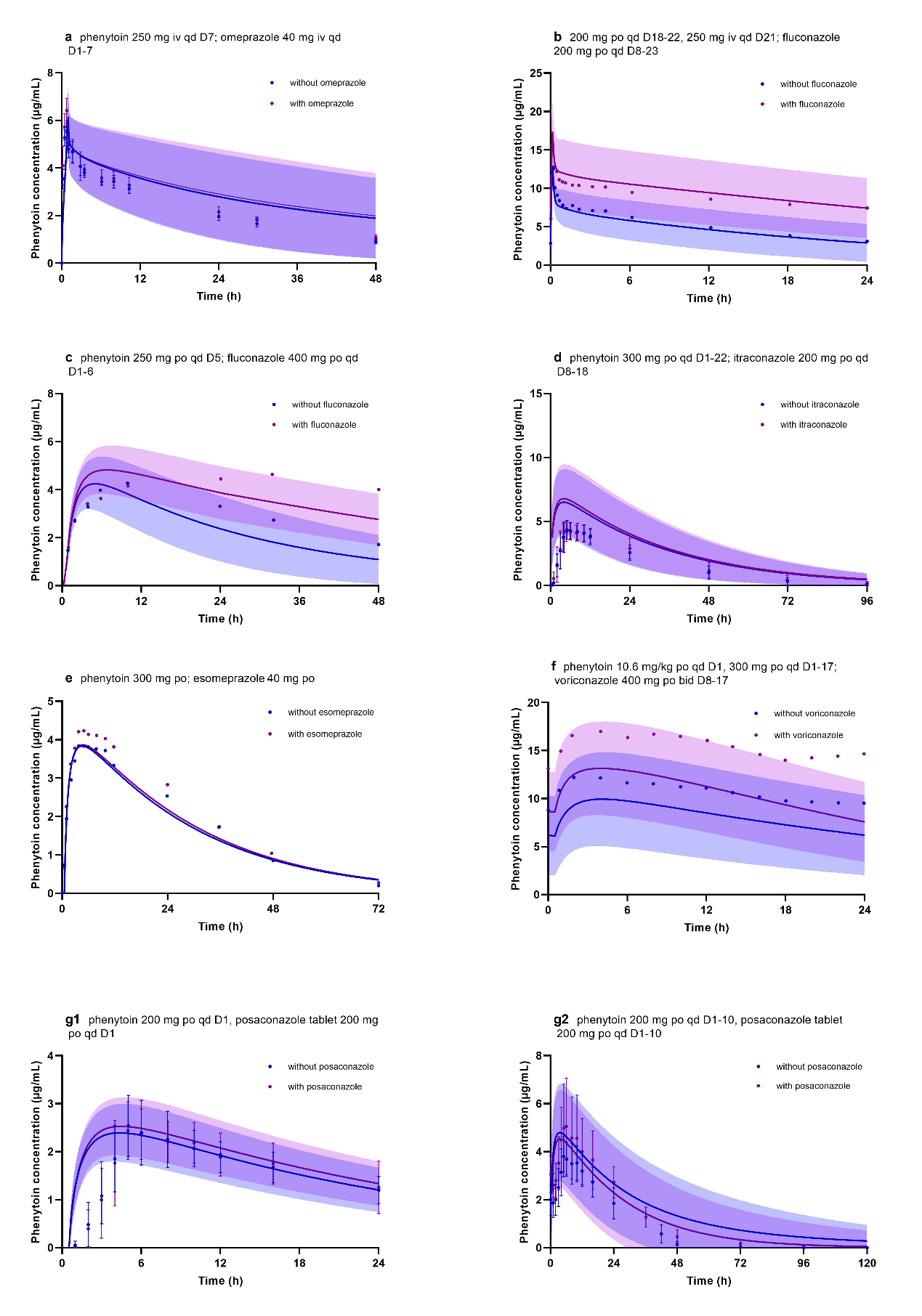


Figure S9. Prediction performance of phenytoin DDI based on PBPK model.^96-100,139,140^


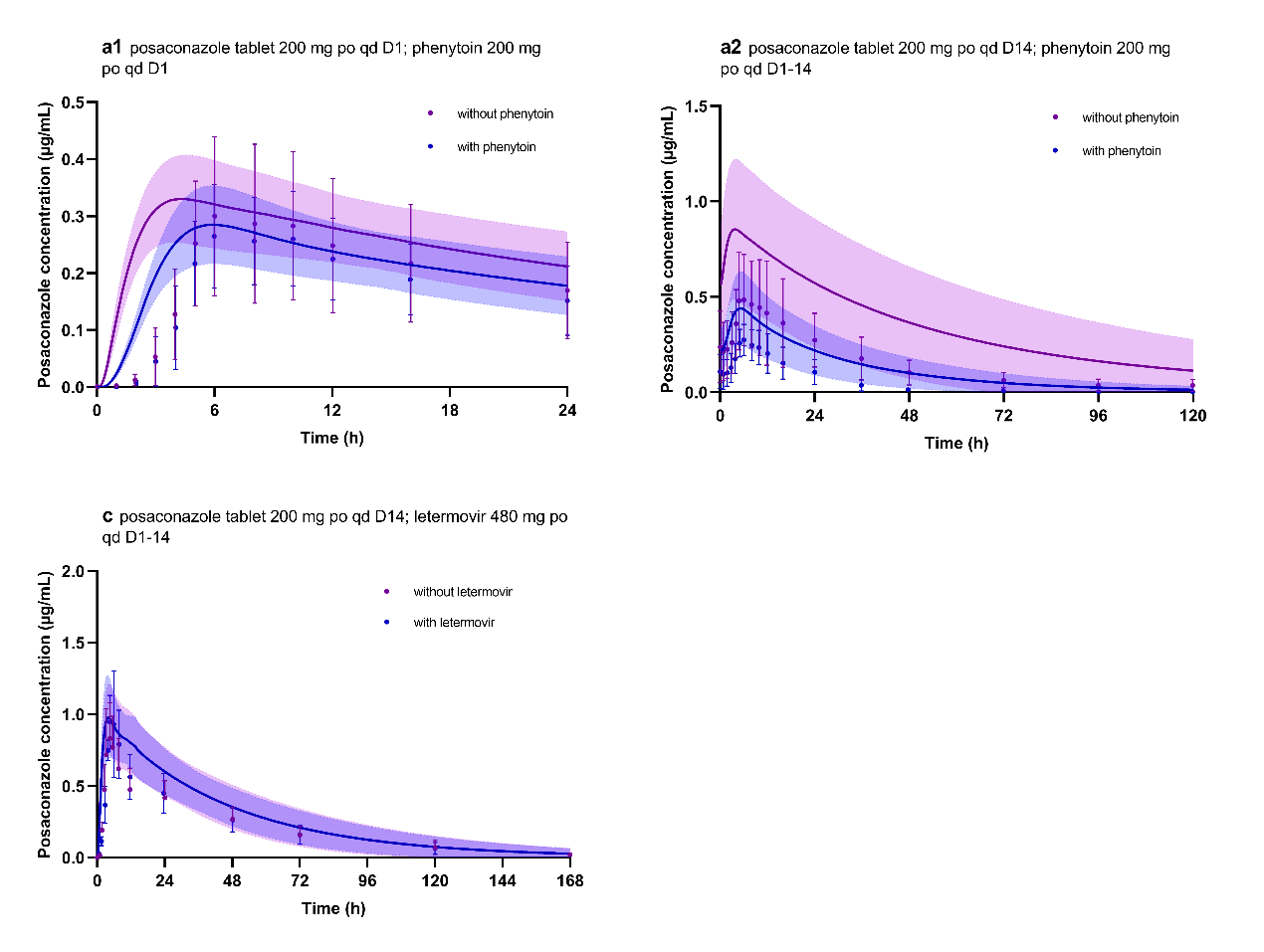


Figure S10. Prediction performance of posaconazole DDI based on PBPK model.^100,133^


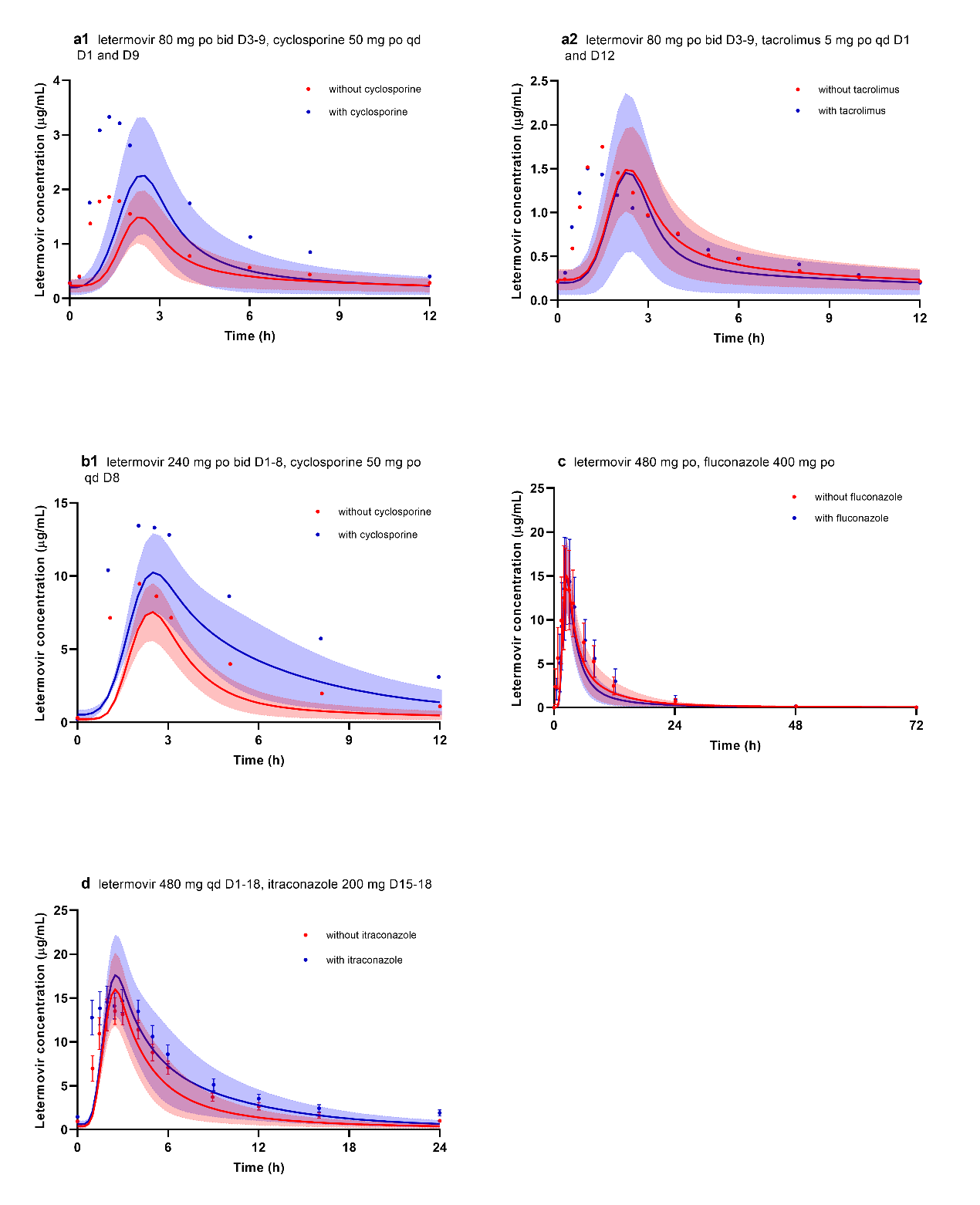


Figure S11. Prediction performance of letermovir DDI based on PBPK model.^106-108,111^


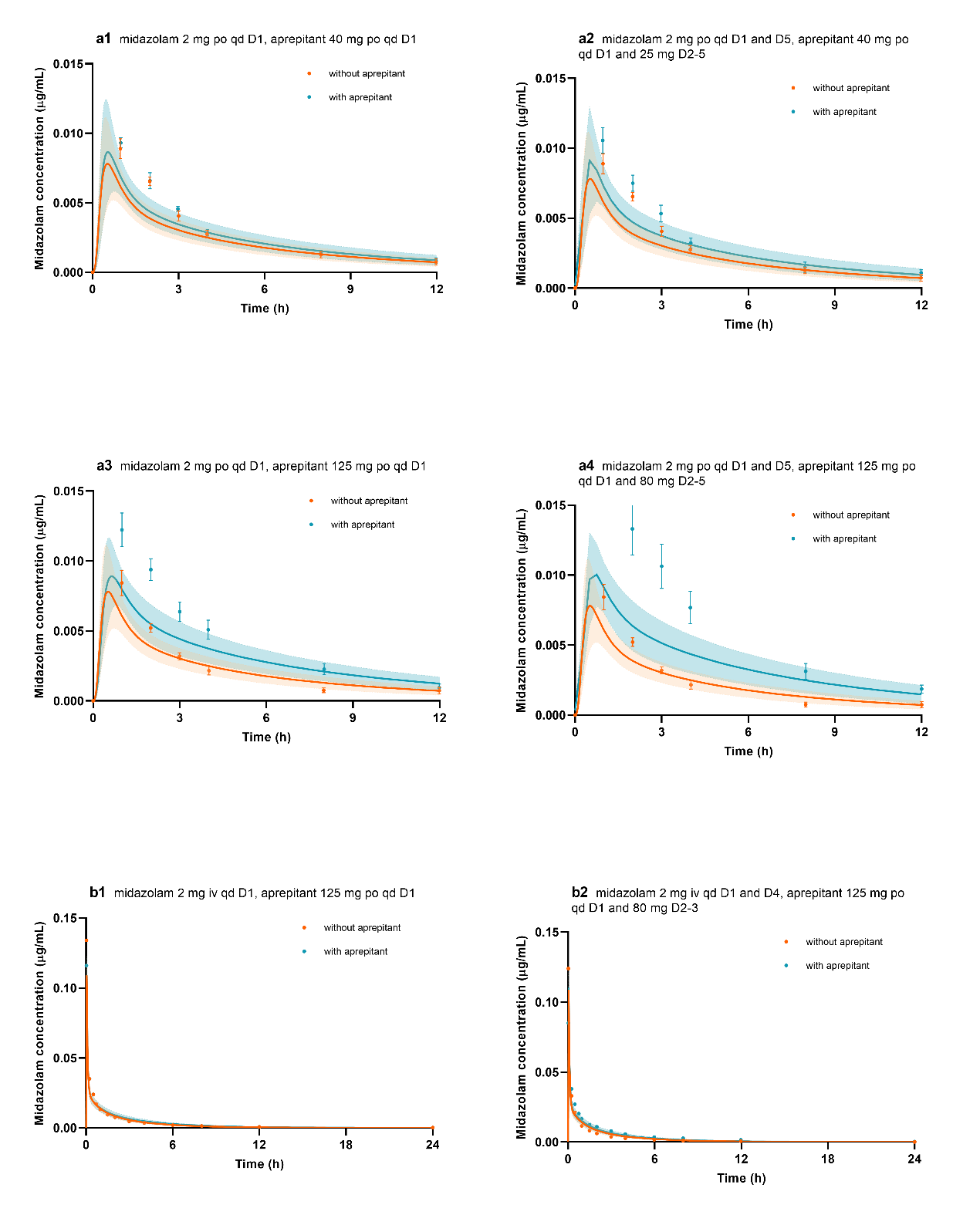


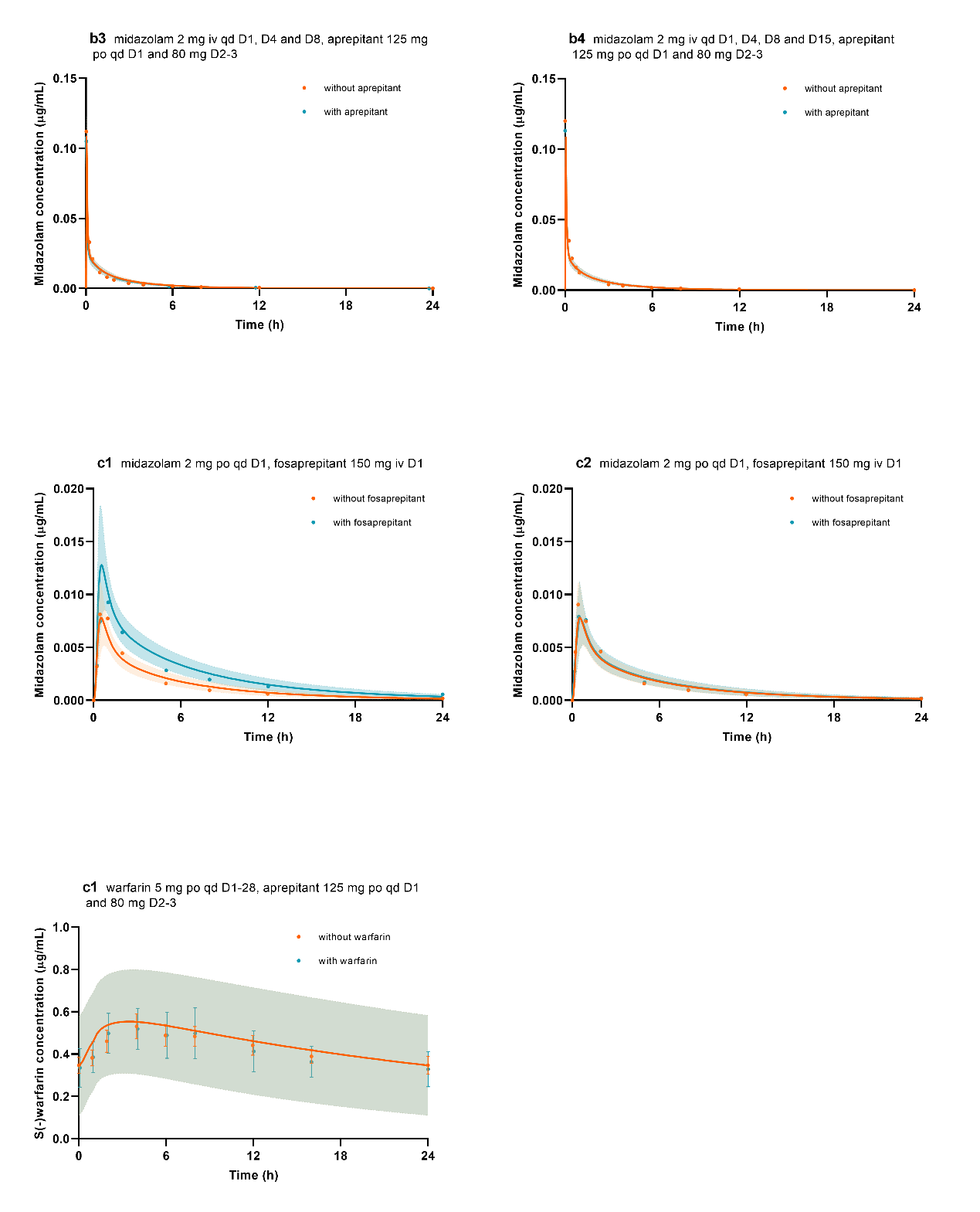


Figure S12. Prediction performance of aprepitant and fosaprepitant DDI based on PBPK model.^135-138^


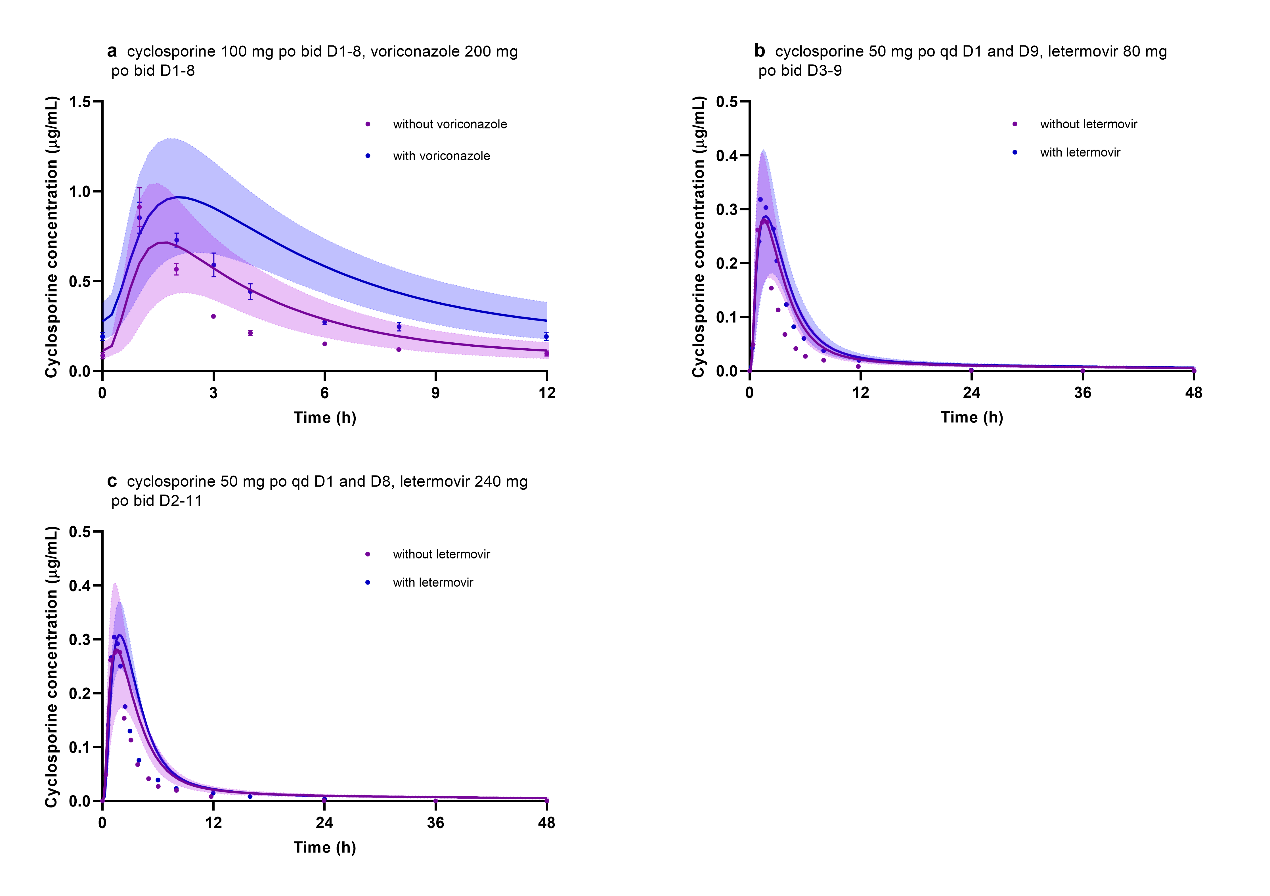


Figure S13. Prediction performance of cyclosporine DDI based on PBPK model.^106,107,120^


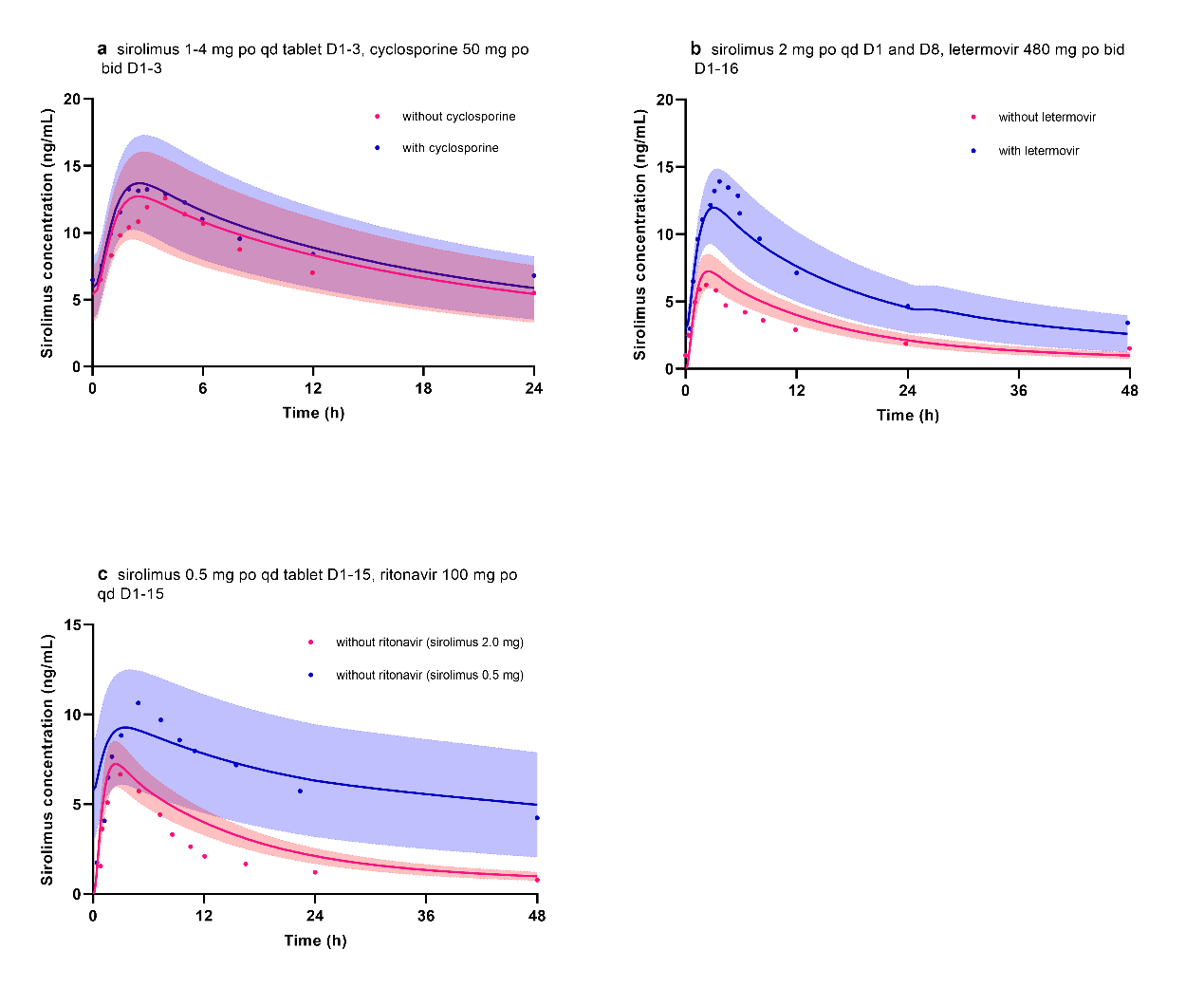


Figure S14. Prediction performance of sirolimus DDI based on PBPK model.^107,127,128^

Reference

1. Diestelhorst C, Boos J, McCune JS, Russell J, Kangarloo SB, Hempel G. Physiologically based pharmacokinetic modelling of Busulfan: a new approach to describe and predict the pharmacokinetics in adults. Cancer Chemother Pharmacol. 2013;72:991-1000.

2. Diestelhorst C, Boos J, McCune JS, Russell J, Kangarloo SB, Hempel G. Predictive performance of a physiologically based pharmacokinetic model of busulfan in children. Pediatr Hematol Oncol. 2014;31:731-742.

3. Ben Hassine K, Seydoux C, Khier S, et al. Pharmacokinetic modeling and simulation with pharmacogenetic insights support the relevance of therapeutic drug monitoring for myeloablative busulfan dosing in adult HSCT. Transplant Cell Ther. 2024;30:332.e331-332.e315.

4. Chiang PC, Wong H. Incorporation of physiologically based pharmacokinetic modeling in the evaluation of solubility requirements for the salt selection process: a case study using phenytoin. AAPS J. 2013;15:1109-1118.

5. Ke AB, Nallani SC, Zhao P, Rostami-Hodjegan A, Unadkat JD. Expansion of a PBPK model to predict disposition in pregnant women of drugs cleared via multiple CYP enzymes, including CYP2B6, CYP2C9 and CYP2C19. Br J Clin Pharmacol. 2013;77:554-570.

6. Abduljalil K, Jamei M, Rostami-Hodjegan A, Johnson TN. Changes in individual drug-independent system parameters during virtual paediatric pharmacokinetic trials: introducing time-varying physiology into a paediatric PBPK model. AAPS J. 2014;16:568-576.

7. Batchelor H, Appleton R, Hawcutt DB. Comparing paediatric intravenous phenytoin doses using physiologically based pharmacokinetic (PBPK) modelling software. Seizure. 2015;33:8-12.

8. Rodriguez-Vera L, Yin X, Almoslem M, et al. Comprehensive physiologically based pharmacokinetic model to assess drug-drug interactions of phenytoin. Pharmaceutics. 2023;15:2486.

9. Li X, Frechen S, Moj D, et al. A physiologically based pharmacokinetic model of voriconazole integrating time-dependent inhibition of CYP3A4, genetic polymorphisms of CYP2C19 and predictions of drug-drug interactions. Clin Pharmacokinet 2020;59:781-808.

10. Zhang Y, Zhao S, Wang C, Zhou P, Zhai S. Application of a physiologically based pharmacokinetic model to characterize time-dependent metabolism of voriconazole in children and support dose optimization. Front Pharmacol. 2021;12:636097.

11. Zubiaur P, Kneller LA, Ochoa D, et al. Evaluation of voriconazole CYP2C19 phenotype-guided dose adjustments by physiologically based pharmacokinetic modeling. Clin Pharmacokinet. 2021;60:261-270.

12. Dong J, Liu SB, Rasheduzzaman JM, Huang CR, Miao LY. Development of physiology Based pharmacokinetic model to predict the drug interactions of voriconazole and venetoclax. Pharm Res. 2022;39:1921-1933.

13. Gong F, Hu H, Ouyang Y, et al. Physiologically-based pharmacokinetic modeling-guided rational combination of tacrolimus and voriconazole in patients with different CYP3A5 and CYP2C19 alleles. Toxicol Appl Pharmacol. 2023;466:116475.

14. Abdullah-Koolmees H, van den Nieuwendijk JF, Hoope S, et al. Whole body physiologically based pharmacokinetic model to explain a patient with drug-drug interaction between voriconazole and flucloxacillin. Eur J Drug Metab Pharmacokinet. 2024;49:689-699.

15. Dong L, Zhuang X, Yang T, Yan K, Cai Y. A physiologically based pharmacokinetic model of voriconazole in human CNS-Integrating time-dependent inhibition of CYP3A4, genetic polymorphisms of CYP2C19 and possible transporter mechanisms. Int J Antimicrob Agents. 2024;64:107310.

16. Saleh A, Schulz J, Schlender JF, et al. Understanding voriconazole metabolism: a middle-Out physiologically-based pharmacokinetic modelling framework integrating in vitro and clinical insights. Clin Pharmacokinet. 2024;63:1609-1630.

17. Wang P, Liu S, Yang J. Physiologically based pharmacokinetic modeling to investigate the disease-drug-drug interactions between voriconazole and nirmatrelvir/ritonavir in COVID-19 patients with CYP2C19 phenotypes. Clin Pharmacol Ther. 2024;116:363-371.

18. Zhao YC, Zhang YK, Gao W, et al. A preliminary exploration of liver microsomes and PBPK to uncover the impact of CYP3A4/5 and CYP2C19 on tacrolimus and voriconazole drug-drug interactions. Sci Rep. 2025;15:6389.

19. Cristofoletti R, Patel N, Dressman JB. Assessment of bioequivalence of weak base formulations under various dosing conditions using physiologically based pharmacokinetic simulations in virtual populations. case examples: ketoconazole and posaconazole. J Pharm Sci. 2017;106:560-569.

20. Hens B, Talattof A, Paixão P, et al. Measuring the impact of gastrointestinal variables on the systemic outcome of two suspensions of posaconazole by a PBPK model. AAPS J. 2018;20:57.

21. Bhatnagar S, Mukherjee D, Salem AH, Miles D, Menon RM, Gibbs JP. Dose adjustment of venetoclax when co-administered with posaconazole: clinical drug-drug interaction predictions using a PBPK approach. Cancer Chemoth Pharm. 2021;87:465-474.

22. Gerner B, Aghai-Trommeschlaeger F, Kraus S, et al. A physiologically-based pharmacokinetic model of ruxolitinib and posaconazole to predict CYP3A4-mediated drug-drug interaction frequently observed in graft versus host disease patients. Pharmaceutics. 2022;14:2556.

23. Malik P, Mian P. Physiologically based pharmacokinetic modeling to refine dosing of posaconazole in young children. Clin Ther. 2025;47:261-270.

24. Vossen M, Sevestre M, Niederalt C, Jang IJ, Willmann S, Edginton AN. Dynamically simulating the interaction of midazolam and the CYP3A4 inhibitor itraconazole using individual coupled whole-body physiologically-based pharmacokinetic (WB-PBPK) models. Theor Biol Med Model. 2007;4:13.

25. Chen Y, Ma F, Lu T, et al. Development of a physiologically based pharmacokinetic model for itraconazole pharmacokinetics and drug-drug interaction prediction. Clin Pharmacokinet. 2016;55:735-749.

26. Hanke N, Frechen S, Moj D, et al. PBPK Models for CYP3A4 and P-gp DDI Prediction: A Modeling Network of Rifampicin, Itraconazole, Clarithromycin, Midazolam, Alfentanil, and Digoxin. CPT Pharmacometrics Syst Pharmacol. 2018;7:647-659.

27. Prieto Garcia L, Janzén D, Kanebratt KP, Ericsson H, Lennernäs H, Lundahl A. Physiologically based pharmacokinetic model of itraconazole and two of its metabolites to improve the predictions and the mechanistic understanding of CYP3A4 drug-drug interactions. Drug Metab Dispos. 2018;46:1420-1433.

28. Türk D, Hanke N, Wolf S, et al. Physiologically based pharmacokinetic models for prediction of complex CYP2C8 and OATP1B1 (SLCO1B1) drug-drug-gene interactions: a modeling network of gemfibrozil, repaglinide, pioglitazone, rifampicin, clarithromycin and itraconazole. Clin Pharmacokinet. 2019;58:1595-1607.

29. Riddell K, Patel A, Collins G, et al. An adaptive physiologically based pharmacokinetic-driven design to investigate the effect of itraconazole and rifampicin on the pharmacokinetics of molibresib (GSK525762) in healthy female volunteers. J Clin Pharmacol. 2021;61:125-137.

30. Yang E, Yu KS, Lee S. Prediction of gastric pH-mediated drug exposure using physiologically-based pharmacokinetic modeling: A case study of itraconazole. CPT Pharmacometrics Syst Pharmacol. 2023;12:865-877.

31. Bergagnini-Kolev M, Kane K, Templeton IE, Curran AK. Evaluation of the potential for drug-drug interactions with inhaled itraconazole using physiologically based pharmacokinetic modelling, based on phase 1 clinical data. AAPS J. 2023;25:62.

32. Li L, Zhang YY, Sharma J, et al. Drug-drug interaction study with itraconazole supplemented with physiologically based pharmacokinetic modelling to characterize the effect of CYP3A inhibitors on venglustat pharmacokinetics. Br J Clin Pharmacol. 2025online.

33. Watt KM, Cohen-Wolkowiez M, Barrett JS, et al. Physiologically based pharmacokinetic approach to determine dosing on extracorporeal life support: fluconazole in children on ECMO. CPT Pharmacometrics Syst Pharmacol. 2018;7:629-637.

34. Gerhart JG, Watt KM, Edginton A, et al. Physiologically-based pharmacokinetic modeling of fluconazole using plasma and cerebrospinal fluid samples From preterm and term infants. CPT Pharmacometrics Syst Pharmacol. 2019;8:500-510.

35. Ogawa SI, Shimizu M, Yamazaki H. Modelled plasma concentrations of pemafibrate with co-administered typical cytochrome P450 inhibitors clopidogrel, fluconazole or clarithromycin predicted by physiologically based pharmacokinetic modelling in virtual populations. Xenobiotica. 2020;50:1413-1422.

36. Salerno SN, Edginton A, Gerhart JG, et al. Physiologically-based pharmacokinetic modeling characterizes the CYP3A-mediated drug-drug interaction between fluconazole and sildenafil in infants. Clin Pharmacol Ther. 2021;109:253-262.

37. Geng K, Shen C, Wang X, et al. A physiologically-based pharmacokinetic/pharmacodynamic modeling approach for drug-drug-gene interaction evaluation of S-warfarin with fluconazole. CPT Pharmacometrics Syst Pharmacol. 2024;13:853-869.

38. Wang YH, Chen D, Hartmann G, Cho CR, Menzel K. PBPK modeling strategy for predicting complex drug interactions of letermovir as a perpetrator in support of product labeling. Clin Pharmacol Ther. 2019;105:515-523.

39. Menzel K, Kuo Y, Chen D, et al. Developing a mechanistic understanding of the nonlinear pharmacokinetics of letermovir and prospective drug interaction with everolimus using physiological-based pharmacokinetic modeling. Clin Transl Sci. 2023;16:1039-1048.

40. Maruyama T, Kasai H, Fukaya Y, Shiokawa M, Kimura T, Hamada Y. Drug-drug interactions between letermovir and tacrolimus in Japanese renal transplant recipients simulated using a physiologically based pharmacokinetic model. Front Microbiol. 2024;15:1480874.

41. Wu F, Gaohua L, Zhao P, et al. Predicting nonlinear pharmacokinetics of omeprazole enantiomers and racemic drug using physiologically based pharmacokinetic modeling and simulation: application to predict drug/genetic interactions. Pharm Res. 2014;31:1919-1929.

42. Feng S, Cleary Y, Parrott N, et al. Evaluating a physiologically based pharmacokinetic model for prediction of omeprazole clearance and assessing ethnic sensitivity in CYP2C19 metabolic pathway. Eur J Clin Pharmacol. 2015;71:617-624.

43. Higashimori M, Shimada H, Ichikawa K, Zhou D. Physiologically based pharmacokinetic modeling to predict exposures in healthy Japanese subjects with different CYP2C19 phenotypes: Esomeprazole case study Int J Clin Pharmacol Ther. 2020;58:29-36.

44. Kanacher T, Lindauer A, Mezzalana E, et al. A physiologically-based pharmacokinetic (PBPK) model network for the prediction of CYP1A2 and CYP2C19 drug-drug-Gene interactions with fuvoxamine, omeprazole, S-mephenytoin, moclobemide, tizanidine, mexiletine, ethinylestradiol, and caffeine. Pharmaceutics. 2020;12:1191.

45. Le Merdy M, Tan ML, Sun D, et al. Physiologically based pharmacokinetic modeling approach to identify the drug-drug interaction mechanism of nifedipine and a proton pump inhibitor, omeprazole. Eur J Drug Metab Pharmacokinet. 2021;46:41-51.

46. Li S, Xie L, Yang L, et al. Prediction of omeprazole pharmacokinetics and its inhibition on gastric acid secretion in humans using physiologically based pharmacokinetic-pharmacodynamic model characterizing CYP2C19 polymorphisms. Pharm Res. 2023;40:1735-1750.

47. Pippa LF, Vozmediano V, Mitrov-Winkelmolen L, et al. Impact of obesity and roux-en-Y gastric bypass on the pharmacokinetics of (R)- and (S)-omeprazole and intragastric pH. CPT Pharmacometrics Syst Pharmacol. 2024;13:1528-1541.

48. Yang R, Lin Y, Chen K, et al. Establishing virtual bioequivalence and clinically relevant specifications for omeprazole enteric-coated capsules by incorporating dissolution data in PBPK modeling. AAPS J. 2024;26:82.

49. Soliman A, Rodriguez-Vera L, Alarcia-Lacalle A, et al. Leveraging omeprazole PBPK/PD modeling to inform drug-drug interactions and specific recommendations for pediatric labeling. Pharmaceutics. 2025;17:373.

50. Gérard C, Bleyzac N, Girard P, Freyer G, Bertrand Y, Tod M. Influence of dosing schedule on organ exposure to cyclosporin in pediatric hematopoietic stem cell transplantation: analysis with a PBPK model. Pharm Res. 2010;27:2602-2613.

51. Gérard C, Bleyzac N, Girard P, Freyer G, Bertrand Y, Tod M. Links between cyclosporin exposure in tissues and graft-versus-host disease in pediatric bone marrow transplantation: analysis by a PBPK model. Pharm Res. 2011;28:531-539.

52. Darwich AS, Pade D, Rowland-Yeo K, et al. Evaluation of an in silico PBPK post-bariatric surgery model through simulating oral drug bioavailability of atorvastatin and cyclosporine. CPT Pharmacometrics Syst Pharmacol. 2013;2:e47.

53. Gertz M, Cartwright CM, Hobbs MJ, et al. Cyclosporine inhibition of hepatic and intestinal CYP3A4, uptake and efflux transporters: application of PBPK modeling in the assessment of drug-drug interaction potential. Pharm Res. 2013;30:761-780.

54. Thiel C, Schneckener S, Krauss M, et al. A systematic evaluation of the use of physiologically based pharmacokinetic modeling for cross-species extrapolation. J Pharm Sci. 2015;104:191-206.

55. Yoshikado T, Yoshida K, Kotani N, et al. Quantitative analyses of hepatic OATP-mediated interactions between statins and inhibitors using PBPK modeling with a parameter optimization method. Clin Pharmacol Ther. 2016;100:513-523.

56. Kim SJ, Toshimoto K, Yao Y, Yoshikado T, Sugiyama Y. Quantitative analysis of complex drug-drug interactions between repaglinide and cyclosporin A/gemfibrozil using physiologically based pharmacokinetic models with in vitro transporter/enzyme inhibition data. J Pharm Sci. 2017;106:2715-2726.

57. Fan J, Chen L, Lu X, Li M, Zhu L. The pharmacokinetic prediction of cyclosporin A after coadministration with Wuzhi capsule. AAPS PharmSciTech. 2019;20:247.

58. Yoon S, Yi S, Rhee SJ, et al. Development of a physiologically-based pharmacokinetic model for cyclosporine in Asian children with renal impairment. Transl Clin Pharmacol. 2019;27:107-114.

59. Yang Y, Li P, Zhang Z, Wang Z, Liu L, Liu X. Prediction of cyclosporin-mediated drug interaction using physiologically based pharmacokinetic model characterizing interplay of drug transporters and enzymes. Int J Mol Sci. 2020;21:7023.

60. Park JE, Shitara Y, Lee W, et al. Improved prediction of the drug-drug interactions of pemafibrate caused by cyclosporine A and rifampicin via PBPK modeling: consideration of the albumin-mediated hepatic uptake of pemafibrate and inhibition constants with preincubation against OATP1B. J Pharm Sci. 2021;110:517-528.

61. Zapke SE, Willmann S, Grebe SO, Menke K, Thürmann PA, Schmiedl S. Comparing predictions of a PBPK model for cyclosporine with drug levels from therapeutic drug monitoring. Front Pharmacol. 2021;12:630904.

62. Schaller S, Michon I, Baier V, Martins FS, Nolain P, Taneja A. Evaluation of BCRP-related DDIs between methotrexate and cyclosporin A using physiologically based pharmacokinetic modelling. Drugs R D. 2024online.

63. Gérard C, Stocco J, Hulin A, et al. Determination of the most influential sources of variability in tacrolimus trough blood concentrations in adult liver transplant recipients: a bottom-up approach. AAPS J. 2014;16:379-391.

64. Purohit HS, Trasi NS, Sun DD, et al. Investigating the impact of drug crystallinity in amorphous tacrolimus capsules on pharmacokinetics and bioequivalence using discriminatory in vitro dissolution testing and physiologically based pharmacokinetic modeling and simulation. J Pharm Sci. 2018;107:1330-1341.

65. Zhang H, Bu F, Li L, et al. Prediction of drug-drug interaction between tacrolimus and principal ingredients of Wuzhi capsule in Chinese healthy volunteers using physiologically-based pharmacokinetic modelling. Basic Clin Pharmacol Toxicol. 2018;122:331-340.

66. Emoto C, Johnson TN, Hahn D, et al. A theoretical physiologically-based pharmacokinetic approach to ascertain covariates explaining the large interpatient variability in tacrolimus disposition. CPT Pharmacometrics Syst Pharmacol. 2019;8:273-284.

67. He Q, Bu F, Zhang H, et al. Investigation of the impact of CYP3A5 polymorphism on drug-drug interaction between tacrolimus and schisantherin A/schisandrin A based on physiologically-based pharmacokinetic modeling. Pharmaceuticals (Basel). 2021;14:198.

68. He Q, Bu F, Wang Q, et al. Examination of the impact of CYP3A4/5 on drug-drug interaction between schizandrol A/schizandrol B and tacrolimus (FK-506): a physiologically based pharmacokinetic modeling approach. Int J Mol Sci. 2022;23:4485.

69. Zhao X, Lu X, Zuo M, et al. Drug-drug interaction comparison between tacrolimus and phenobarbital in different formulations for paediatrics and adults. Xenobiotica. 2021;51:877-884.

70. Itohara K, Yano I, Nakagawa S, et al. Extrapolation of physiologically based pharmacokinetic model for tacrolimus from renal to liver transplant patients. Drug Metab Pharmacokinet. 2022;42:100423.

71. Cai L, Ke M, Wang H, et al. Physiologically based pharmacokinetic model combined with reverse dose method to study the nephrotoxic tolerance dose of tacrolimus. Arch Toxicol. 2023;97:2659-2673.

72. Loer HLH, Feick D, Rüdesheim S, et al. Physiologically based pharmacokinetic modeling of tacrolimus for food-drug and CYP3A drug-drug-gene interaction predictions. CPT Pharmacometrics Syst Pharmacol. 2023;12:724-738.

73. El-Khateeb E, Chinnadurai R, Al Qassabi J, et al. Using prior knowledge on systems through PBPK to gain further insight into routine clinical data on trough concentrations: the case of tacrolimus in chronic kidney disease. Ther Drug Monit. 2023;45:743-753.

74. Hong E, Carmanov E, Shi A, et al. Application of physiologically based pharmacokinetic modeling to predict drug-drug interactions between elexacaftor/tezacaftor/ivacaftor and tacrolimus in lung transplant recipients. Pharmaceutics. 2023;15:1438.

75. Pei L, Li R, Zhou H, et al. A physiologically based pharmacokinetic approach to recommend an individual dose of tacrolimus in adult heart transplant recipients. Pharmaceutics. 2023;15:2580.

76. Van der Veken M, Brouwers J, Ozbey AC, et al. Investigating tacrolimus disposition in paediatric patients with a physiologically based pharmacokinetic model incorporating CYP3A4 ontogeny, mechanistic absorption and red blood cell binding. Pharmaceutics. 2023;15:2231.

77. Guan Y, Liu X, Huang K, et al. Physiologically-based pharmacokinetic modelling to investigate the effect of CYP3A4/3A5 maturation on tacrolimus pharmacokinetics in paediatric HSCT patients. Eur J Pharm Sci. 2024;201:106839.

78. Martischang R, Nikolaou A, Daali Y, Samer CF, Terrier J. Guidance on selecting optimal steady-state tacrolimus concentrations for continuous IV perfusion: insights from physiologically based pharmacokinetic modeling. Pharmaceuticals (Basel). 2024;17:1047.

79. Karakitsios E, Angelerou MF, Kapralos I, Tsakiridou G, Kalantzi L, Dokoumetzidis A. Integrating in vitro dissolution and physiologically based pharmacokinetic modeling for generic drug development: evaluation of amorphous solid dispersion formulations for tacrolimus. Pharmaceutics. 2025;17:227.

80. Xu J, Guo G, Zhou S, et al. Physiologically-based pharmacokinetic modeling to predict the exposure and provide dosage regimens of tacrolimus in pregnant women with infection disease. Eur J Pharm Sci. 2025;206:107003.

81. Emoto C, Fukuda T, Cox S, Christians U, Vinks AA. Development of a physiologically-based Pharmacokinetic model for sirolimus: predicting bioavailability based on intestinal CYP3A content. CPT Pharmacometrics Syst Pharmacol. 2013;2:e59.

82. Emoto C, Fukuda T, Johnson TN, Adams DM, Vinks AA. Development of a pediatric physiologically based pharmacokinetic model for sirolimus: Applying principles of growth and maturation in neonates and infants. CPT Pharmacometrics Syst Pharmacol. 2015;4:e17.

83. Emoto C, Fukuda T, Venkatasubramanian R, Vinks AA. The impact of CYP3A5*3 polymorphism on sirolimus pharmacokinetics: insights from predictions with a physiologically-based pharmacokinetic model. Br J Clin Pharmacol. 2015;80:1438-1446.

84. Chen KF, Jones HM. PBPK perspective on alternative CYP3A4 inducers for rifampin. CPT Pharmacometrics Syst Pharmacol. 2022;11:1543-1546.

85. Sanchez RI, Wang RW, Newton DJ, et al. Cytochrome P450 3A4 is the major enzyme involved in the metabolism of the substance P receptor antagonist aprepitant. Drug Metab Dispos. 2004;32:1287-1292.

86. Thiel C, Cordes H, Fabbri L, et al. A comparative analysis of drug-induced hepatotoxicity in clinically relevant situations. PLoS Comput Biol. 2017;13:e1005280.

87. Ehrsson H, Hassan M, Ehrnebo M, Beran M. Busulfan kinetics. Clin Pharmacol Ther. 1983;34:86-89.

88. Grochow LB, Jones RJ, Brundrett RB, et al. Pharmacokinetics of busulfan: correlation with veno-occlusive disease in patients undergoing bone marrow transplantation. Cancer Chemother Pharmacol. 1989;25:55-61.

89. Hassan M, Oberg G, Ehrsson H, et al. Pharmacokinetic and metabolic studies of high-dose busulphan in adults. Eur J Clin Pharmacol. 1989;36:525-530.

90. Hassan M, Oberg G, Bekassy AN, et al. Pharmacokinetics of high-dose busulphan in relation to age and chronopharmacology. Cancer Chemoth Pharm. 1991;28:130-134.

91. Fernandez HF, Tran HT, Albrecht F, Lennon S, Caldera H, Goodman MS. Evaluation of safety and pharmacokinetics of administering intravenous busulfan in a twice-daily or daily schedule to patients with advanced hematologic malignant disease undergoing stem cell transplantation. Biol Blood Marrow Transplant. 2002;8:486-492.

92. Xie HL, Wu XM, Zhuang BY, et al. Limited sampling strategies for clinical plasma concentration monitoring of busulfan in hematopoietic stem cell transplantation patients. Chin Pharm J. 2015;50:1424-1429.

93. Kikuchi T, Mori T, Ohwada C, et al. Pharmacokinetics of intravenous busulfan as condition for hematopoietic stem cell transplantation: comparison between combinations with cyclophosphamide and fludarabine. Int J Hematol. 2021;113:128-133.

94. Gugler R, Manion CV, Azarnoff DL. Phenytoin: pharmacokinetics and bioavailability. Clin Pharmacol Ther. 1976;19:135-142.

95. Randinitis EJ, Buchanan RA, Kinkel AW. Pharmacokinetic profile of a 300-mg extended phenytoin sodium capsule (Dilantin) formulation. Epilepsia. 1990;31:458-464.

96. Blum RA, Wilton JH, Hilligoss DM, et al. Effect of fluconazole on the disposition of phenytoin. Clin Pharmacol Ther. 1991;49:420-425.

97. Touchette MA, Chandrasekar PH, Milad MA, Edwards DJ. Contrasting effects of fluconazole and ketoconazole on phenytoin and testosterone disposition in man. Br J Clin Pharmacol. 1992;34:75-78.

98. Ducharme MP, Slaughter RL, Warbasse LH, et al. Itraconazole and hydroxyitraconazole serum concentrations are reduced more than tenfold by phenytoin. Clin Pharmacol Ther. 1995;58:617-624.

99. Purkins L, Wood N, Ghahramani P, Love ER, Eve MD, Fielding A. Coadministration of voriconazole and phenytoin: pharmacokinetic interaction, safety, and toleration. Br J Clin Pharmacol. 2003;56 Suppl 1:37-44.

100. Krishna G, Sansone-Parsons A, Kantesaria B. Drug interaction assessment following concomitant administration of posaconazole and phenytoin in healthy men. Curr Med Res Opin. 2007;23:1415-1422.

101. Ezzet F, Wexler D, Courtney R, Krishna G, Lim J, Laughlin M. Oral bioavailability of posaconazole in fasted healthy subjects: comparison between three regimens and basis for clinical dosage recommendations. Clin Pharmacokinet. 2005;44:211-220.

102. Kersemaekers WM, van Iersel T, Nassander U, et al. Pharmacokinetics and safety study of posaconazole intravenous solution administered peripherally to healthy subjects. Antimicrob Agents Chemother. 2015;59:1246-1251.

103. Li H, Wei Y, Zhang S, et al. Pharmacokinetics and safety of posaconazole administered by intravenous solution and oral tablet in healthy Chinese subjects and effect of food on tablet bioavailability. Clin Drug Investig. 2019;39:1109-1116.

104. Krishna G, Ma L, Martinho M, O'Mara E. Single-dose phase I study to evaluate the pharmacokinetics of posaconazole in new tablet and capsule formulations relative to oral suspension. Antimicrob Agents Chemother. 2012;56:4196-4201.

105. Krishna G, Ma L, Martinho M, Preston RA, O'Mara E. A new solid oral tablet formulation of posaconazole: a randomized clinical trial to investigate rising single- and multiple-dose pharmacokinetics and safety in healthy volunteers. J Antimicrob Chemother. 2012;67:2725-2730.

106. Kropeit D, von Richter O, Stobernack HP, Rübsamen-Schaeff H, Zimmermann H. Pharmacokinetics and safety of letermovir coadministered with cyclosporine A or tacrolimus in healthy subjects. Clin Pharmacol Drug Dev. 2018;7:9-21.

107. McCrea JB, Macha S, Adedoyin A, et al. Pharmacokinetic drug-drug interactions between letermovir and the immunosuppressants cyclosporine, tacrolimus, sirolimus, and mycophenolate mofetil. J Clin Pharmacol. 2019;59:1331-1339.

108. Adedoyin A, Fancourt C, Menzel K, et al. Assessment of pharmacokinetic interaction between letermovir and fluconazole in healthy participants. Clin Pharmacol Drug Dev. 2021;10:198-206.

109. Menzel K, Kothare P, McCrea JB, Chu X, Kropeit D. Absorption, metabolism, distribution, and excretion of letermovir. Curr Drug Metab. 2021;22:784-794.

110. Asari K, Ishii M, Yoshitsugu H, et al. Pharmacokinetics, safety, and tolerability of letermovir following single- and multiple-dose administration in healthy Japanese subjects. Clin Pharmacol Drug Dev. 2022;11:938-948.

111. McCrea JB, Menzel K, Fancourt C, et al. Evaluation of the inhibitory effects of itraconazole on letermovir. Br J Clin Pharmacol. 2023;89:2122-2130.

112. CINVANTI® (aprepitant) injectable emulsion, for intravenous use. 2003; https://www.accessdata.fda.gov/drugsatfda_docs/label/2017/209296s000lbl.pdf.

113. Lasseter KC, Gambale J, Jin B, et al. Tolerability of fosaprepitant and bioequivalency to aprepitant in healthy subjects. J Clin Pharmacol. 2007;47:834-840.

114. Shadle CR, Murphy MG, Liu Y, et al. A single-dose bioequivalence and food effect study with aprepitant and fosaprepitant dimeglumine in healthy young adult subjects. Clin Pharmacol Drug Dev. 2012;1:93-101.

115. Ottoboni T, Keller MR, Cravets M, Clendeninn N, Quart B. Bioequivalence of HTX-019 (aprepitant IV) and fosaprepitant in healthy subjects: a Phase I, open-label, randomized, two-way crossover evaluation. Drug Des Devel Ther. 2018;12:429-435.

116. Lu K, Lin S, Wang Y, et al. Pharmacokinetics and safety of fosaprepitant dimeglumine in healthy Chinese volunteers: bioequivalence study. Clin Pharmacol Drug Dev. 2021;10:748-755.

117. Ducharme MP, Warbasse LH, Edwards DJ. Disposition of intravenous and oral cyclosporine after administration with grapefruit juice. Clin Pharmacol Ther. 1995;57:485-491.

118. Elder CA, Moore M, Chang CT, et al. Efficacy and pharmacokinetics of two formulations of cyclosporine A in patients with psoriasis. J Clin Pharmacol. 1995;35:865-875.

119. Stein CM, Sadeque AJ, Murray JJ, Wandel C, Kim RB, Wood AJ. Cyclosporine pharmacokinetics and pharmacodynamics in African American and white subjects. Clin Pharmacol Ther. 2001;69:317-323.

120. Romero AJ, Le Pogamp P, Nilsson LG, Wood N. Effect of voriconazole on the pharmacokinetics of cyclosporine in renal transplant patients. Clin Pharmacol Ther. 2002;71:226-234.

121. Bergman AJ, Burke J, Larson P, et al. Effects of ezetimibe on cyclosporine pharmacokinetics in healthy subjects. J Clin Pharmacol. 2006;46:321-327.

122. Budde K, Lehne G, Winkler M, et al. Influence of everolimus on steady-state pharmacokinetics of cyclosporine in maintenance renal transplant patients. J Clin Pharmacol. 2005;45:781-791.

123. Li F, Lin H, Feng S, et al. A phase I, single-sequence, open-label study to evaluate the drug-drug interaction between hetrombopag and cyclosporine in healthy Chinese subjects. Br J Clin Pharmacol. 2023;89:2160-2167.

124. NEORAL® oral solution (cyclosporine oral solution, USP) MODIFIED. 2009; https://www.accessdata.fda.gov/drugsatfda_docs/label/2009/050715s027,050716s028lbl.pdf.

125. Brattström C, Säwe J, Jansson B, et al. Pharmacokinetics and safety of single oral doses of sirolimus (rapamycin) in healthy male volunteers. Ther Drug Monit. 2000;22:537-544.

126. Leelahavanichkul A, Areepium N, Vadcharavivad S, et al. Pharmacokinetics of sirolimus in Thai healthy volunteers. J Med Assoc Thai. 2005;88 Suppl 4:S157-S162.

127. Wang HF, Qiu F, Wu X, et al. Steady-state pharmacokinetics of sirolimus in stable adult Chinese renal transplant patients. Clin Pharmacol Drug Dev. 2014;3:235-241.

128. Zha J, Jiang Q, Yao BB, Cohen DE, Carter DC, Menon RM. Effects of a ritonavir-containing regimen on the pharmacokinetics of sirolimus or everolimus in healthy adult subjects. Pharmacol Res Perspect. 2022;10:e01024.

129. Kelly PA, Napoli K, Kahan BD. Conversion from liquid to solid rapamycin formulations in stable renal allograft transplant recipients. Biopharm Drug Dispos. 1999;20:249-253.

130. RAPAMUNE (sirolimus) oral solution RAPAMUNE (sirolimus) tablets, for oral use. 2017; https://www.accessdata.fda.gov/drugsatfda_docs/label/2017/021083s059,021110s076lbl.pdf.

131. Leung LY, Lim HK, Abell MW, Zimmerman JJ. Pharmacokinetics and metabolic disposition of sirolimus in healthy male volunteers after a single oral dose. Ther Drug Monit. 2006;28:51-61.

132. Hassan M, Oberg G, Björkholm M, Wallin I, Lindgren M. Influence of prophylactic anticonvulsant therapy on high-dose busulphan kinetics. Cancer Chemoth Pharm. 1993;33:181-186.

133. Marshall WL, McCrea JB, Macha S, et al. Pharmacokinetics and tolerability of letermovir coadministered with azole antifungals (posaconazole or voriconazole) in healthy subjects. J Clin Pharmacol. 2018;58:897-904.

134. Kropeit D, McCormick D, Erb-Zohar K, Stobernack HP, Zimmermann H, Rübsamen-Schaeff H. Pharmacokinetics and safety of letermovir and midazolam coadministration in healthy subjects. Clin Pharmacol Drug Dev. 2022;11:16-24.

135. Majumdar AK, McCrea JB, Panebianco DL, et al. Effects of aprepitant on cytochrome P450 3A4 activity using midazolam as a probe. Clin Pharmacol Ther. 2003;74:150-156.

136. Shadle CR, Lee Y, Majumdar AK, et al. Evaluation of potential inductive effects of aprepitant on cytochrome P450 3A4 and 2C9 activity. J Clin Pharmacol. 2004;44:215-223.

137. Marbury TC, Ngo PL, Shadle CR, et al. Pharmacokinetics of oral dexamethasone and midazolam when administered with single-dose intravenous 150 mg fosaprepitant in healthy adult subjects. J Clin Pharmacol. 2011;51:1712-1720.

138. Depré M, Van Hecken A, Oeyen M, et al. Effect of aprepitant on the pharmacokinetics and pharmacodynamics of warfarin. Eur J Clin Pharmacol. 2005;61:341-346.

139. Gugler R, Jensen JC. Omeprazole inhibits oxidative drug metabolism. Studies with diazepam and phenytoin in vivo and 7-ethoxycoumarin in vitro. Gastroenterology. 1985;89:1235-1241.

140. Andersson T, Hassan-Alin M, Hasselgren G, Röhss K. Drug interaction studies with esomeprazole, the (S)-isomer of omeprazole. Clin Pharmacokinet. 2001;40:523-537.
